# Computational and experimental mapping of the allosteric network of two manganese ABC transporters

**DOI:** 10.1002/pro.70039

**Published:** 2025-01-29

**Authors:** Ozge Duman, Anastasiya Kuznetsova, Nurit Livnat Levanon, Moti Grupper, Akarun Ayca Ersoy, Burcin Acar, Amit Kessel, Nir Ben‐Tal, Oded Lewinson, Turkan Haliloglu

**Affiliations:** ^1^ Department of Chemical Engineering Bogazici University Istanbul Turkey; ^2^ Polymer Research Center Bogazici University Istanbul Turkey; ^3^ Department of Molecular Microbiology Bruce and Ruth Rappaport Faculty of Medicine, Technion‐Israel Institute of Technology Haifa Israel; ^4^ Infectious Disease Unit Rambam Health Care Campus Haifa Israel; ^5^ School of Neurobiology, Biochemistry and Biophysics, George S. Wise Faculty of Life Sciences Tel‐Aviv University Tel‐Aviv Israel

**Keywords:** ABC transporter, allostery, causal dynamic interactions, elastic network models, MntBC transporter, PsaBC transporter, transfer entropy, transition metal transport

## Abstract

Transition metals (e.g., Fe^2/3+^, Zn^2+^, Mn^2+^) are essential enzymatic cofactors in all organisms. Their environmental scarcity led to the evolution of high‐affinity uptake systems. Our research focuses on two bacterial manganese ABC importers, *Streptococcus pneumoniae* PsaBC and *Bacillus anthracis* MntBC, both critical for virulence. Both importers share a similar homodimeric structure, where each protomer comprises a transmembrane domain (TMD) linked to a cytoplasmic nucleotide‐binding domain (NBD). Due to their size and slow turnover rates, the utility of conventional molecular simulation approaches to reveal functional dynamics is limited. Thus, we employed a novel, computationally efficient method integrating Gaussian Network Models (GNM) with information theory Transfer Entropy (TE) calculations. Our calculations are in remarkable agreement with previous functional studies. Furthermore, based on the calculations, we generated 10 point‐mutations and experimentally tested their effects, finding excellent concordance between computational predictions and experimental results. We identified “allosteric hotspots” in both transporters, in the transmembrane translocation pathway, at the coupling helices linking the TMDs and NBDs, and in the ATP binding sites. In both PsaBC and MntBC, we observed bi‐directional information flow between the two TMDs, with minimal allosteric transmission to the NBDs. Conversely, the NBDs exhibited almost no NBD‐NBD allosteric crosstalk but showed pronounced information flow from the NBD of one protomer towards the TMD of the other protomer. This unique allosteric “footprint” distinguishes ABC importers of transition metals from other members of the ABC transporter superfamily establishing them as a distinct functional class. This study offers the first comprehensive insight into the conformational dynamics of these vital virulence determinants, providing potential avenues for developing urgently needed novel antibacterial agents.

## 1 INTRODUCTION

ATP‐binding cassette (ABC) transporters comprise one of the largest and oldest protein families of any proteome, with ~120 members in higher plants, 49 in human, and ~80 in prokaryotes. They harness the energy of ATP hydrolysis to move molecules across cell membranes (Ames et al., 1992; Higgins, 1992). In humans, ABC transporters protect cells from toxic materials and deliver biomolecules to designated compartments (Bordignon et al., 2010). They are directly linked to human diseases and to tumor chemotherapy resistance (Kerppola et al., 1991; Lanfermeijer et al., 1999). In prokaryotes, the high‐affinity acquisition of nutrients is mediated by ABC transporters that function as importers (hereafter ABC importers). Each ABC importer is highly specific to a single nutrient or a group of highly similar nutrients. Under conditions of nutrient limitation, such as those encountered during host infection, their role is underscored. It is therefore not surprising that a large number of bacterial ABC importers have been identified as key virulence determinants (Mattle et al., 2010; Thomas & Tampé, 2020).

The functional unit of an ABC transporter minimally consists of four domains: two transmembrane domains (TMDs) that form the translocation pathway and two cytoplasmic nucleotide‐binding domains (NBDs) that provide energy via ATP hydrolysis. ABC importers additionally require a substrate binding protein (SBP) which binds the substrate and delivers it to its cognate transporter (Andreini, 2012; Thomas et al., 2020).

Recent studies have identified two distinct groups among ABC importers: Type‐I and Type‐II. The Type‐I group includes importers for peptides, amino acids, and sugars, while the Type‐II group specializes in importing rare micronutrients such as siderophores and vitamins. These groups differ significantly in their structure, mechanisms, and conformational dynamics (Lewinson & Livnat‐Levanon, 2017). Type‐I importers are substrate‐regulated systems. In the absence of substrate, they exhibit low basal ATP hydrolysis, which is markedly stimulated upon substrate binding. The cyclic association and dissociation between the substrate‐binding protein (SBP) and the transporter, as well as the conformational dynamics required for transport, are all enhanced by the presence of the substrate. In contrast, Type‐II importers operate differently. These systems are largely substrate‐insensitive, with significant ATP hydrolysis occurring even in the absence of substrate and minimal stimulation observed when it is present. Furthermore, the interactions between the SBP and the transporter, as well as the conformational cycle, are primarily driven by ATP rather than the substrate.

Among ABC importers, the subgroup responsible for importing transition metals remains the least characterized. It is currently unclear whether this subgroup belongs to Type‐I, Type‐II, or represents an entirely different subgroup.

Transition metals such as Cu^1/2+^, Zn^2+^, Mn^2+^, and Fe^2/3+^, serve as indispensable micronutrients for all life forms (Andreini, 2012; Andreini et al., 2008). They cannot be synthesized and therefore must be acquired from the environment. Given the environmental scarcity of transition metals, high‐affinity transport systems play a crucial role in their acquisition (Ammendola et al., 2007; Chandrangsu et al., 2017). In bacteria, acquisition of essential transition metals is primarily mediated by ABC transporters (Klein & Lewinson, 2011). The absolute necessity for these metals positions them at the nexus of host‐pathogen interactions (Ammendola et al., 2007; Bearden & Perry, 1999; Gat et al., 2005; Marra et al., 2002; McDevitt et al., 2011; Paik et al., 2003; Pattery et al., 1999; Remy et al., 2013; Rodriguez & Smith, 2006). In the host environment, transition metals are scarce, with their availability further constrained by several innate immunity mechanisms (Becker & Skaar, 2014; Murdoch & Skaar, 2022). In adapting to this metal‐deprived environment, bacteria rely on their ABC transporters importers. Consequently, it is unsurprising that ABC importers of transition metals have emerged as pivotal determinants of bacterial virulence (Ammendola et al., 2007; Bearden & Perry, 1999; Gat et al., 2005; McDevitt et al., 2011; Paik et al., 2003; Remy et al., 2013).

Two notable examples are demonstrated by the manganese importers PsaBCA and MntBCA of *Streptococcus pneumonia* and *Bacillus anthracis*, respectively. The crucial role of these ABC importers is emphasized by the complete loss of virulence observed upon their inactivation (Berry & Paton, 1996; Gat et al., 2005; Tseng et al., 2002). One mechanistic aspect of ABC transporters of transition metals that remains unexplored is their conformational dynamics: what are the conformational changes that underlie the transport cycle, how they are modulated by binding of ligands, and how are distant protein domains connected allosterically.

The experimental exploration of proteins' conformational dynamics is highly challenging and often requires single molecule techniques (Yang et al., 2018). This is particularly true for membrane proteins like ABC transporters. Molecular simulations offer an alternative approach, but the transport cycle, ATPase cycle, and conformational changes of ABC transporters occur on the order of many milliseconds to seconds (Goudsmits et al., 2017; Yang et al., 2018), making conventional molecular dynamics impractical. To address this challenge, we utilized a computationally efficient approach that integrates the Gaussian Network Model (GNM) and Transfer Entropy (TE) formalism (Altintel et al., 2022; Ersoy et al., 2023; Haliloglu et al., 1997).

GNM simplifies the intricate protein polymer into a network of interconnected nodes and springs, where each node represents a main chain Cα atom, and the springs denote harmonic interactions between Cα atoms within a specified cutoff distance. This simplified model facilitates the computation of accessible motions, referred to as GNM “modes”, inherent to the protein's structure and topology. The collective ensemble of these GNM modes defines the conformational space accessible to the protein (Bahar et al., 1997; Haliloglu et al., 1997).

GNM can be enhanced through integration with transfer entropy (TE), an information theory metric quantifying the information exchange between two processes (Hacisuleyman & Erman, 2017). The combination of GNM and TE, denoted as GNM‐TE, enables the comprehensive exploration of proteins' allosteric interactions governing conformational changes and identification of residues or domains exhibiting synchronous movements (Altintel et al., 2022; Bahar et al., 1997; Hacisuleyman & Erman, 2017).

In the context of GNM‐TE, each allosterically coupled residue pair provides insights into causality, elucidating which residue (or domain) serves as the driving or leading event, and which acts as the following or driven event. The synergy of GNM and TE can yield a conformational allosteric dynamics “footprint” for any protein with a known structure.

Here, we combine GNM‐TE and experiments to investigate the allosteric connectivity driving conformational changes in the ABC transporters/virulence determinants PsaBCA and MntBCA. We identified the residues that serve as the main sources of allosteric control and found them predominantly positioned along the transmembrane translocation pathway, at the lipid‐TMD interface, at the coupling helices, and adjacent to the ATP binding sites. The computational identification of allosteric “hotspots” was validated through mutational analysis, revealing excellent correspondence between the two. This demonstrates the efficacy and accuracy of the GNM‐TE method. Furthermore, the results reveal a unique pattern of allosteric interactions between the transmembrane domains (TMDs) and nucleotide binding domains (NBDs), a pattern not yet observed in other ABC transporters. Collectively, this study demonstrates that although ABC transporters of transition metals are structurally similar to other ABC transporters, their allosteric characteristics are very different.

## 2 RESULTS

### 2.1 Allosteric hotspots in *Streptococcus pneumonia* PsaBC

In previous study (Acar et al., 2020), we found that Type‐I and Type‐II ABC importers exhibit markedly different conformational dynamics. In Type‐I systems, which transport peptides, amino acids, and sugars, the substrate plays a dominant role in controlling conformational dynamics, with the flow of allosteric information directed from the extracellular to the intracellular side. Conversely, in Type‐II systems, which transport siderophores and vitamins, ATP has the predominant role in regulating conformational dynamics. In these systems, the flow of allosteric information is directed from the intracellular to the extracellular side. These two subgroups have distinct “footprints” of conformational allosteric dynamics.

To acquire such a “footprint” of *Streptococcus pneumonia* PsaBC (see Neville et al., 2021), GNM‐TE analysis was conducted utilizing the published inward‐facing conformation of the importer (PDB ID 7KYP Neville et al., 2021). Initially, we considered the 10 slowest GNM modes, comprehensively representing the full dynamic spectrum (Figure S1).

**FIGURE 1:**
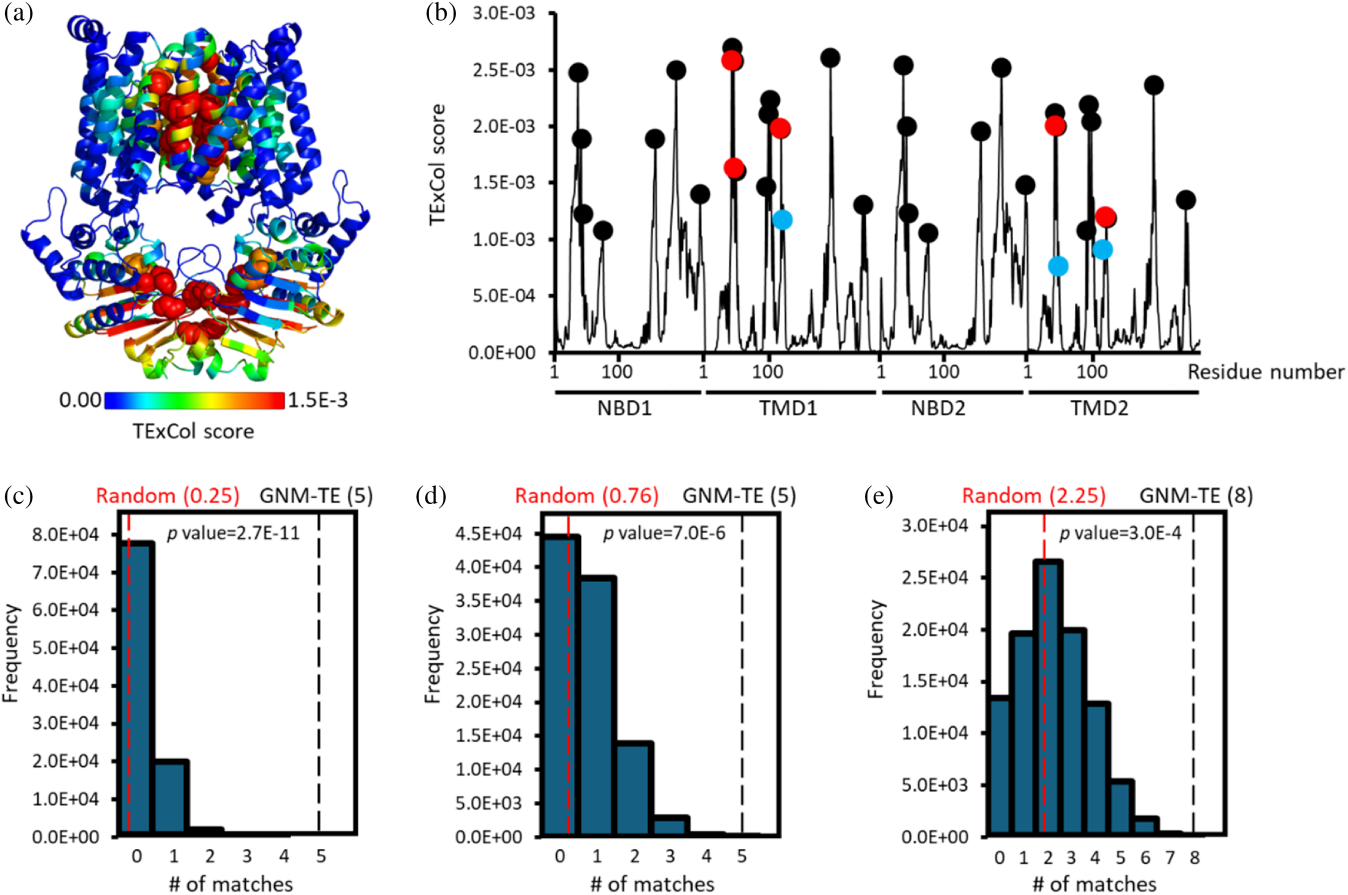
Identification of allosteric hotspots in PsaBC. (a) Residues of PsaBC (PDB ID: 7KYP) are color‐coded based on their allosteric output (expressed in terms of TE × Col scores), with blue and red denoting low and high values, respectively. Residues identified as allosteric peaks are depicted as spheres. (b) Shown are the TE × Col scores for all residues of PsaBC (black trace). The 31 positions classified as allosteric peaks are indicated by circles. Red and cyan circles signify allosteric peaks that precisely match or are within <7 Å from the positions of functionally essential residues, respectively. (c–e) 100,000 sets of 31 randomized positions were generated and for each such set the number of matches with the positions of the 8 essential was counted. Shown is the probability distribution when considering only exact matches (c), or also first‐coordination sphere interactions within a cutoff distance of ≤4 Å (d), or also second‐coordination sphere interactions within a cutoff distance of ≤7 Å (e). Red and black dashed vertical lines indicate the mean value of matches for the random and GNM‐TE based predictions, respectively. Also shown are the p values obtained by a one‐tailed hypothesis tests with a significance level of 0.05.

In this analysis, TE × Col scores were calculated for all residues of PsaBC. TE × Col scores encompass both the net TE values (how much information is sent) and the degree of collectivity of information transfer (how many residues receive the information, see methods for full details). Residues assigned TE × Col scores surpassing a threshold set at 2 times the average TE × Col score and 2 times the average peak prominence were considered allosteric hotspots. Using these criteria, we identified 31 residues that serve as information sources (hereafter allosteric hotspots) located in both the Transmembrane Domains (TMDs) and Nucleotide‐Binding Domains (NBDs) (Figure 1a,b). Within the TMDs, residues assigned the highest GNM‐TE scores are those forming the transmembrane permeation pathway, while in the NBDs, the highest values are associated with residues in closest proximity to the ATP binding sites (Figure 1a).

To assess the fidelity of GNM‐TE predictions, we scrutinized the correspondence between the predicted allosteric hotspots and functionally essential residues in PsaBC. The premise for this analysis is the observation that the positions of allosteric hotspots often coincide with those of functionally essential ones (Altintel et al., 2022; Ersoy et al., 2023). To date, only four residues in PsaBC have been experimentally validated to be essential (Neville et al., 2021). These include Asp46 and His50, responsible for transmembrane coordination of manganese, and gating residues Phe121, Phe124. Given that PsaBC forms a homodimer, each of these residues is duplicated in the assembled transporter, totaling eight essential residues per functional unit. Notably, five out of the eight essential residues precisely aligned with the positions of the GNM‐TE predicted allosteric hotspots (Figure 1b, red spheres).

To ascertain the statistical significance of this co‐localization, we compared it to the probability of randomly identifying the eight essential residues. As previously mentioned, 31 allosteric peaks were identified in PsaBC. Therefore, we generated 100,000 sets of 31 randomly localized allosteric hotspots and tallied how often the positions of functionally essential residues were accurately guessed using this random assignment. As shown in Figure 1c, the accuracy of the GNM‐TE predictions greatly outperforms the random one: Compared to the five correct assignments of the GNM‐TE predictions, the distribution of the random allocations has a mean value of only 0.25 (*p* = 2.7E‐11). Expanding our analysis to include not only exact matches but also first‐ or second‐coordination sphere interactions (i.e., peaks located within <4 or <7 Å from the functionally essential residues), we observed that all eight residues are associated with the positions of GNM‐TE peaks and those of functionally essential residues, greatly surpassing the random identification rate (Figure 1d,e).

**FIGURE 2:**
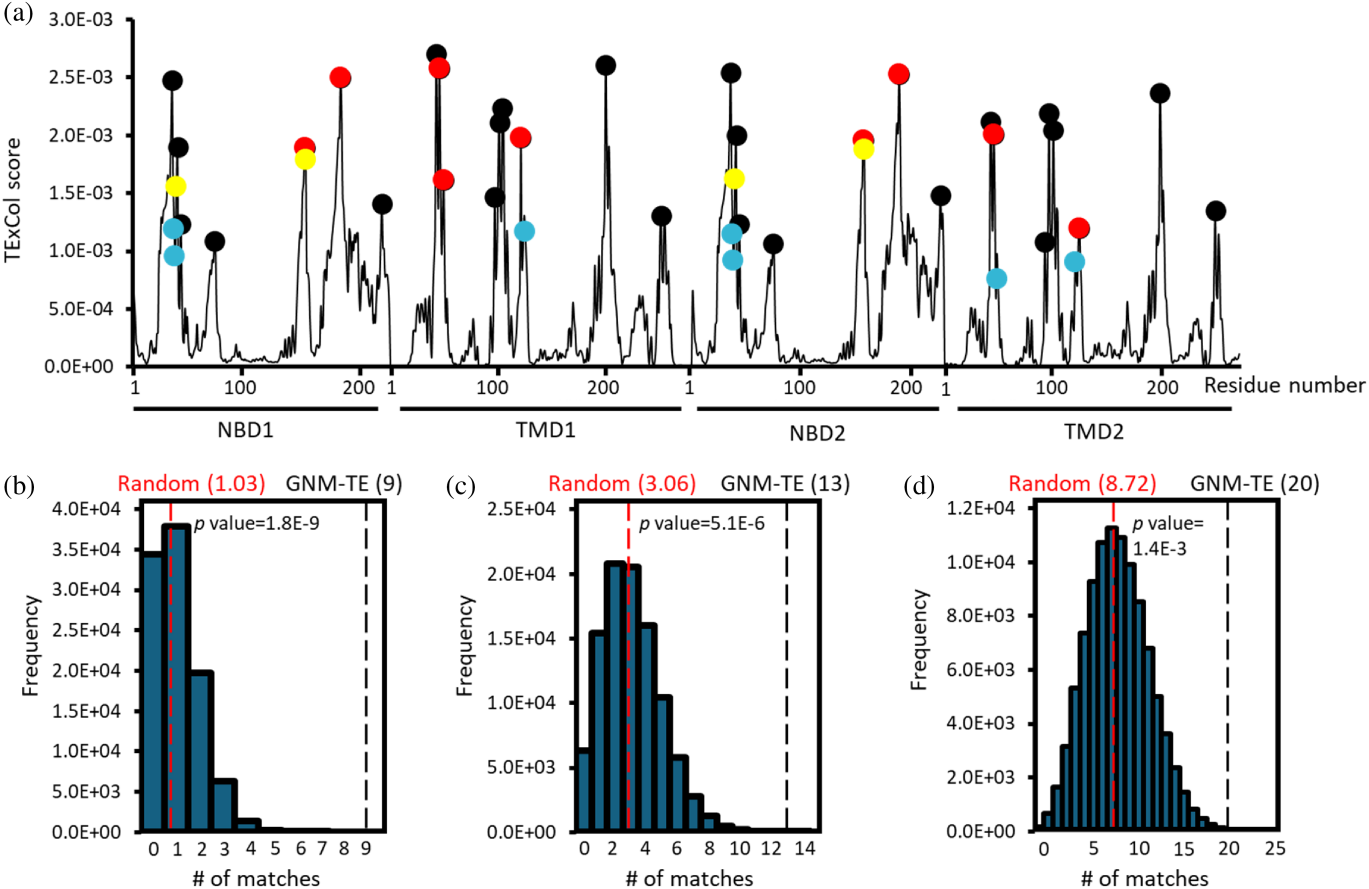
Quality assessment of GNM‐TE with an expanded set of reference residues. (a) Shown are the TE × Col scores for all residues of PsaBC (black trace). The 31 positions classified as allosteric peaks, are indicated by circles. In addition to the eight residues shown to be essential in PsaBC, this expanded set of 34 reference residues includes residues found to be essential in homologous ABC transporters. Allosteric peaks that precisely match positions of residues comprising the expanded set of reference residues are indicated as red circles, and peaks that are within <4 Å or <7 Å are indicated as yellow and cyan circles, respectively. (b–d) 100,000 sets of 31 randomized positions were generated and for each such set the number of matches with the positions of the expanded set of 34 reference residues was counted. Shown is the probability distribution when considering only exact matches (b), or also first‐coordination sphere interactions within a cutoff distance of ≤4 Å (c), or also second‐coordination sphere interactions within a cutoff distance of ≤7 Å (d). Red and black dashed vertical lines indicate the mean value of matches for the random and GNM‐TE based predictions, respectively. Also shown are the *p* values obtained by a one‐tailed hypothesis tests with a significance level of 0.05.

Subsequently, we assembled a second set of reference residues, expanding beyond the experimentally validated eight residues to incorporate additional residues shown to be essential in homologous ABC transporters, resulting in a total of 34 residues (see Table S1). Notably, this expanded set of 34 residues (Table S1) closely aligns with the allosteric peaks identified by GNM‐TE (Figure 2a). This co‐segregation demonstrated superior performance compared to a random allocation of allosteric peaks, whether we considered only exact matches (Figure 2b), or whether we included also first‐ or second‐ coordination sphere interactions (Figure 2c,d). Lastly, we examined a third set of 66 reference residues, which, in addition to the previously considered residues incorporated all residues from the conserved motifs of the ABC transporter superfamily (Table S1). Remarkably, akin to the previous sets of reference residues, the GNM‐TE predictions exhibited significant accuracy compared to random allocation, whether assessing exact matches or first‐ or second‐coordination sphere interactions (Figure S2).

The results presented thus far were obtained using the 10 slowest GNM modes which represent the full dynamic spectrum of PsaBC (Figure S1). However, during the calculations, the large entropic contribution of the slowest modes may overshadow that of the next slowest modes, which might hold significant functional relevance (Goudsmits et al., 2017). To address this issue, one approach is to recompute the analysis while excluding the slowest GNM mode(s). This approach has proven effective in identifying important functional sites within a dataset of allosteric proteins (Altintel et al., 2022) and in the homologous human ABC transporter, CFTR (Ersoy et al., 2023).

**FIGURE 3:**
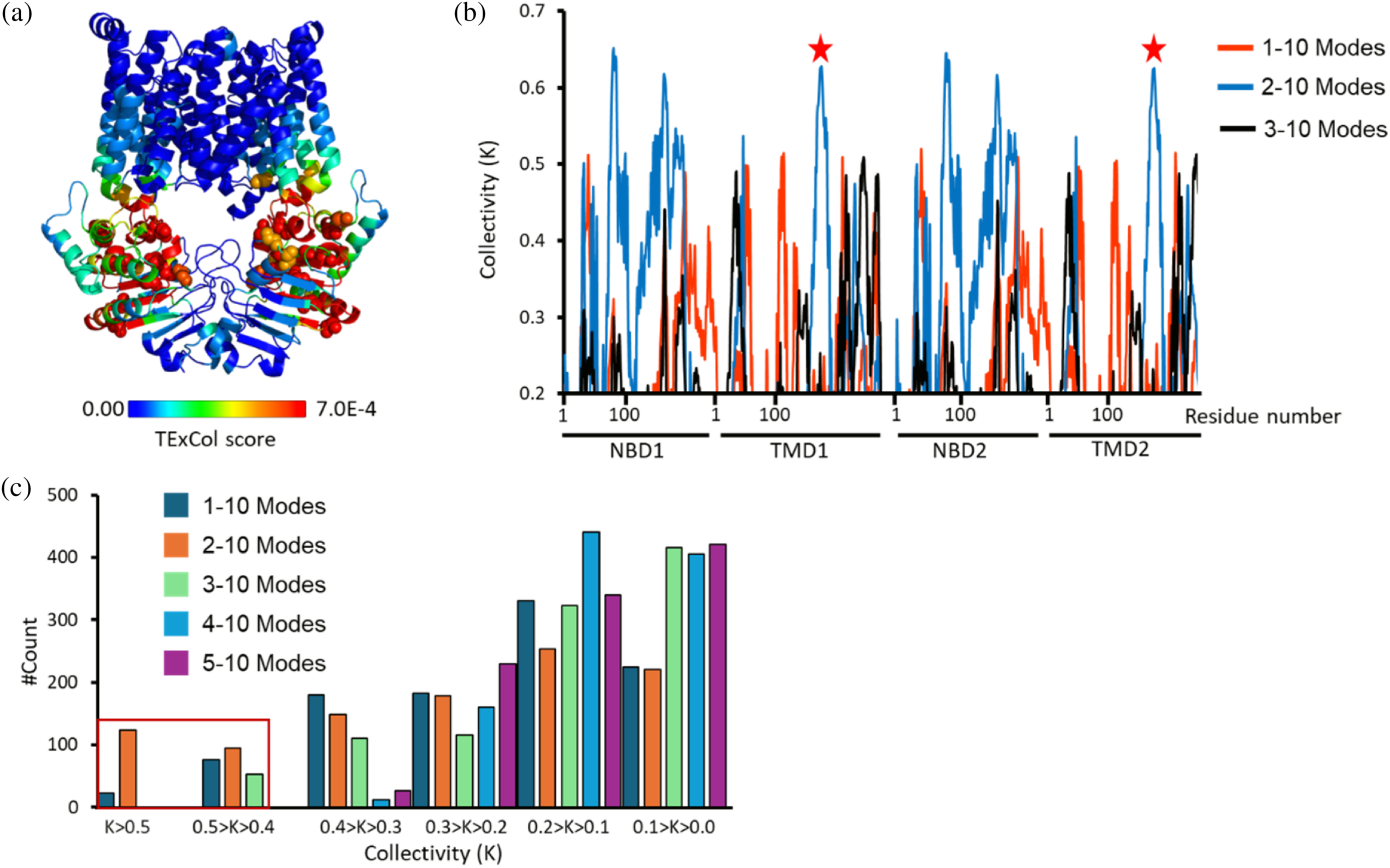
The coupling helices play a major allosteric role. (a) Residues of PsaBC (PDB ID: 7KYP) are color‐coded based on their allosteric output (expressed in terms of TE × Col scores), computed following exclusion of the slowest mode. Blue and red denoting low and high values, respectively, and residues identified as allosteric peaks are depicted as spheres. (b) Shown are the collectivity values (K) for all residues of PsaBC across different mode ranges: 1–10 modes (red), 2–10 modes (blue), and 3–10 modes (black). The red stars indicate the position of the coupling helices. (c) Distribution of residues' collectivity values (K) across different subsets of modes (1–10, 2–10, … 5–10 modes). The two highest collectivity value ranges are highlighted by a red rectangle.

Replication of the calculations after excluding the slowest GNM mode revealed that the coupling helices emerge as prominent allosteric peaks (Figure 3a). Located at the interface between the nucleotide‐binding domains (NBDs) and transmembrane domains (TMDs), the coupling helices are known to play a critical role in allosteric transmission, linking ATP binding and hydrolysis at the NBDs to conformational changes in the TMDs (Acar et al., 2020; Johnson & Chen, 2018; Joseph et al., 2011; Kim & Chen, 2018; Nguyen et al., 2018).

The TE × Col parameter used to measure allosteric importance integrates two components: net transfer entropy (TE), which quantifies the amount of information transmitted by a specific residue, and collectivity (Col), which reflects the number of residues receiving this information. The scale of the TE component is strongly influenced by the subset of modes included in the analysis, with higher absolute values corresponding to slow modes and lower absolute values to fast modes. This dependence complicates the comparison of allosteric importance of residues across calculations performed with different subsets of modes. To enable such comparisons in a scale‐independent manner, we focused on the collectivity (Col) component. Unlike the TE component—and by extension, the TE × Col parameter—the scale of Col is always between 0 and 1, regardless of the GNM modes selected for analysis.

We therefore computed Col values for all residues of PsaBC including in the analysis the first 10 slowest GNM modes (modes 1–10), as well as after excluding the first and second slowest modes (modes 2–10 and 3–10, respectively). Affirming their prominent role in allosteric transduction, the coupling helices were found to exhibit the highest Col values in the 2–10 subset (Figure 3b). Additional residues with elevated Col values were identified within conserved motifs of the ABC transporter superfamily, which are critical for ATP binding and hydrolysis. These include N36 in the Walker A motif, Q76 in the Q‐loop, and L157 in the Walker B motif. Further supporting the allosteric significance of the coupling helices, analysis of the Col value distribution demonstrated that the 1–10, 2–10, and 3–10 subsets are distinct from the rest with residues of high collectivities, and irrespective of the mode subset considered, the Col scores of the coupling helices were among the highest identified (Figure 3c).

### 2.2 Allosteric hotspots in *Bacillus anthracis* MntBC

Encouraged by the successful performance of GNM‐TE in the case of PsaBC, we extended a parallel analysis to the manganese transporter MntBC from *Bacillus anthracis*. PsaBC and MntBC are homologous transporters, with their Nucleotide‐Binding Domains (NBDs) sharing 43.3% sequence identity and 76.7% similarity, and their Transmembrane Domains (TMDs) sharing 55.8% sequence identity and 87.6% similarity. Furthermore, they exhibit common functional attributes, including transport specificity and identical manganese‐coordinating residues (Kuznetsova et al., 2021; Neville et al., 2021). However, in contrast to PsaBC, the structure of MntBC is unknown, posing challenges for structure‐based calculations.

**FIGURE 4:**
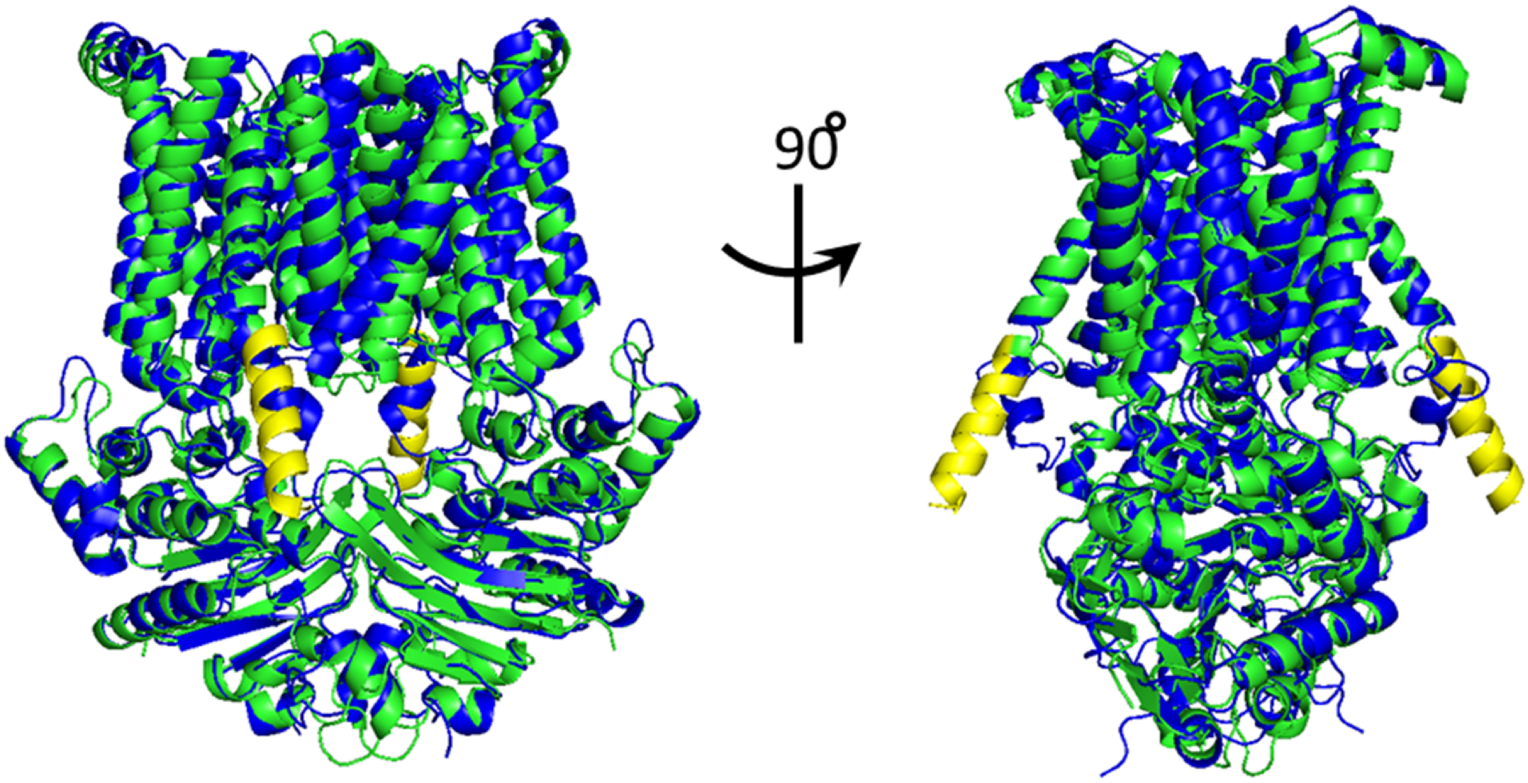
Alternative structural models of MntBC. Structural alignment of the 3D models of *Bacillus anthracis* MntBC generated by AlphaFold2 (green ribbons) or by homology modeling using PsaBC (PDB ID: 7KYP) as a template (blue ribbons). The extended cytosolic alpha‐helix of the AlphaFold2 model is shown in yellow.

We generated two structural models of *Bacillus anthracis* MntBC: a homology model using PsaBC as a template (see methods for details) and an AlphaFold2 (Jumper et al., 2021; Varadi et al., 2022) model. The two models exhibited considerable similarity, with an RMSD of 3.34 Å over 1013 α‐carbons (Figure 4). However, the AlphaFold2 model included several domains of lower certainty, among which was an extended cytosolic alpha‐helix, a feature not previously observed in any ABC transporter (Figure 4, yellow helix). Given these uncertainties, we opted to use the homology model for our computational analysis.

**FIGURE 5:**
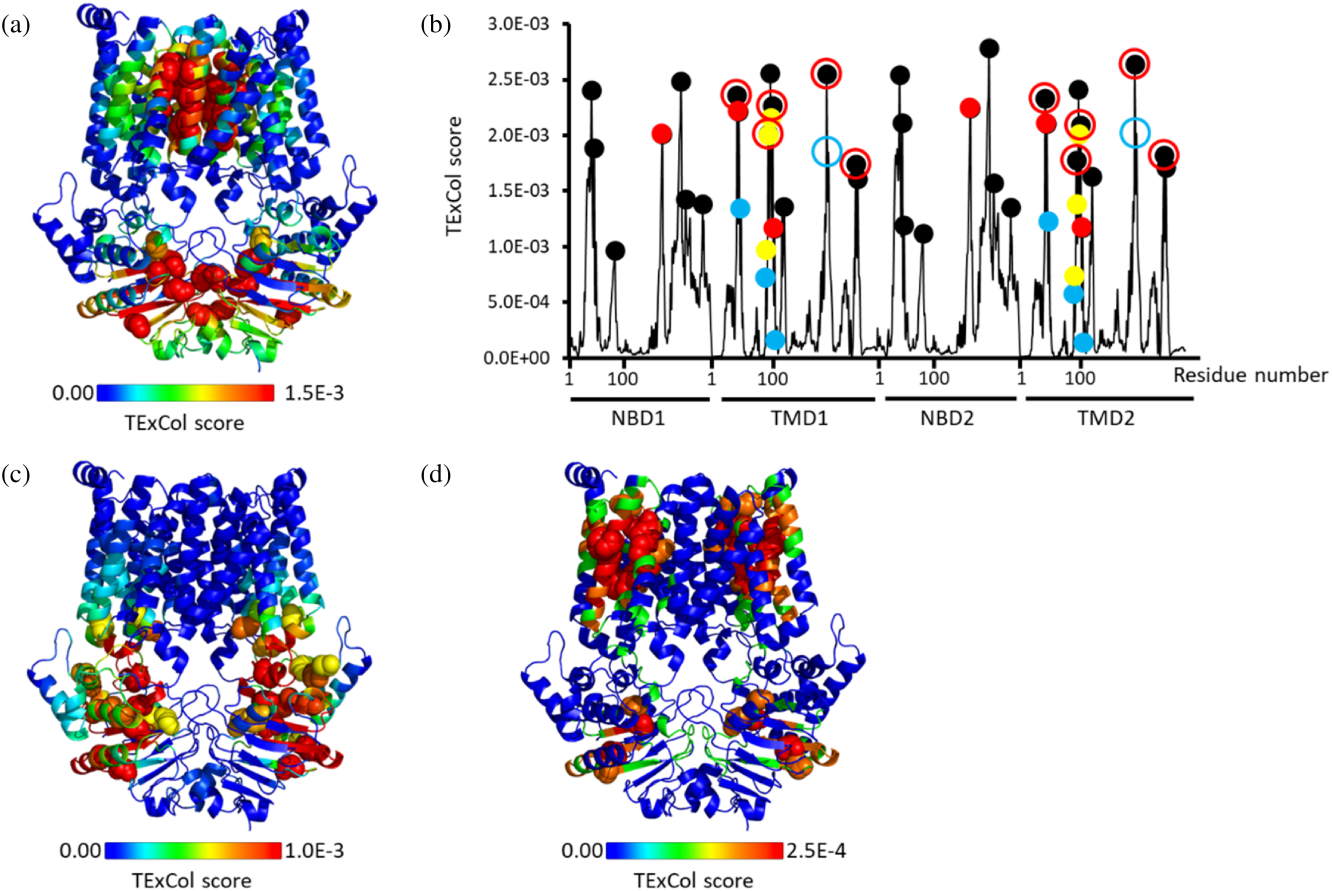
Correlation between allosteric hotspots in MntBC and functionally essential residues. (a) Residues of MntBC are color‐coded based on their allosteric output (expressed in terms of TE × Col scores). Blue and red denoting low and high values, respectively, and residues identified as allosteric peaks are depicted as spheres. (b) Shown are the TE × Col scores for all residues of PsaBC (black trace). The 35 positions classified as allosteric peaks are indicated by circles. Red solid circles indicate allosteric peaks that precisely match the positions of functionally essential residues, and allosteric peaks that are within <4 Å or <7 Å from positions of functionally essential residues are indicated by yellow and cyan solid circles, respectively. Residues not previously identified to be functionally essential yet correspond precisely to a position of an allosteric peak or are found adjacent to one are indicated by open red or cyan circles, respectively. (c) and (d) same as A, only the computations were performed after exclusion of the first slowest mode (c) or the two slowest modes (d).

Applying the same TE × Col score peak criteria utilized for PsaBC, we identified 35 residues acting as allosteric hotspots in the 10 slowest modes of MntBC (Figure 5). Similar to our observations with PsaBC, we noted a remarkable correspondence between the positions of GNM‐TE peaks and residues previously experimentally validated as essential (Kuznetsova et al., 2021), residues demonstrated to be crucial in homologous ABC transporters, and residues from the conserved motifs of the ABC transporter superfamily (Figures 5b, S3–S5, and Table S2).

Notably, akin to the results obtained with PsaBC, the GNM‐TE predictions of MntBC demonstrated significant accuracy compared to random allocation, whether evaluating exact matches or first‐ or second‐coordination sphere interactions (Figures S3–S5).

To identify latent allosteric hotspots, we applied the same methodology described above for the analysis of PsaBC, excluding one or two of the slowest GNM modes. Similar to the results observed for PsaBC, excluding the slowest GNM mode revealed that the coupling helices and their interacting residues in the NBDs are significant allosteric sites (Figure 5c). When the two slowest GNM modes were excluded, distinct allosteric peaks emerged at the interfaces between the NBDs and TMDs, as well as in two symmetric clusters of TMD helices (Figure 5d). These clusters are centrally positioned within the plasma membrane region and adjacent to the protein‐lipid interface. This finding suggests that, while the protein core predominantly mediates allosteric communication (Figure 5a), the peripheral regions also play a critical role in transmitting allosteric signals (Figure 5d).

### 2.3 Experimental verification of computational predictions

**FIGURE 6:**
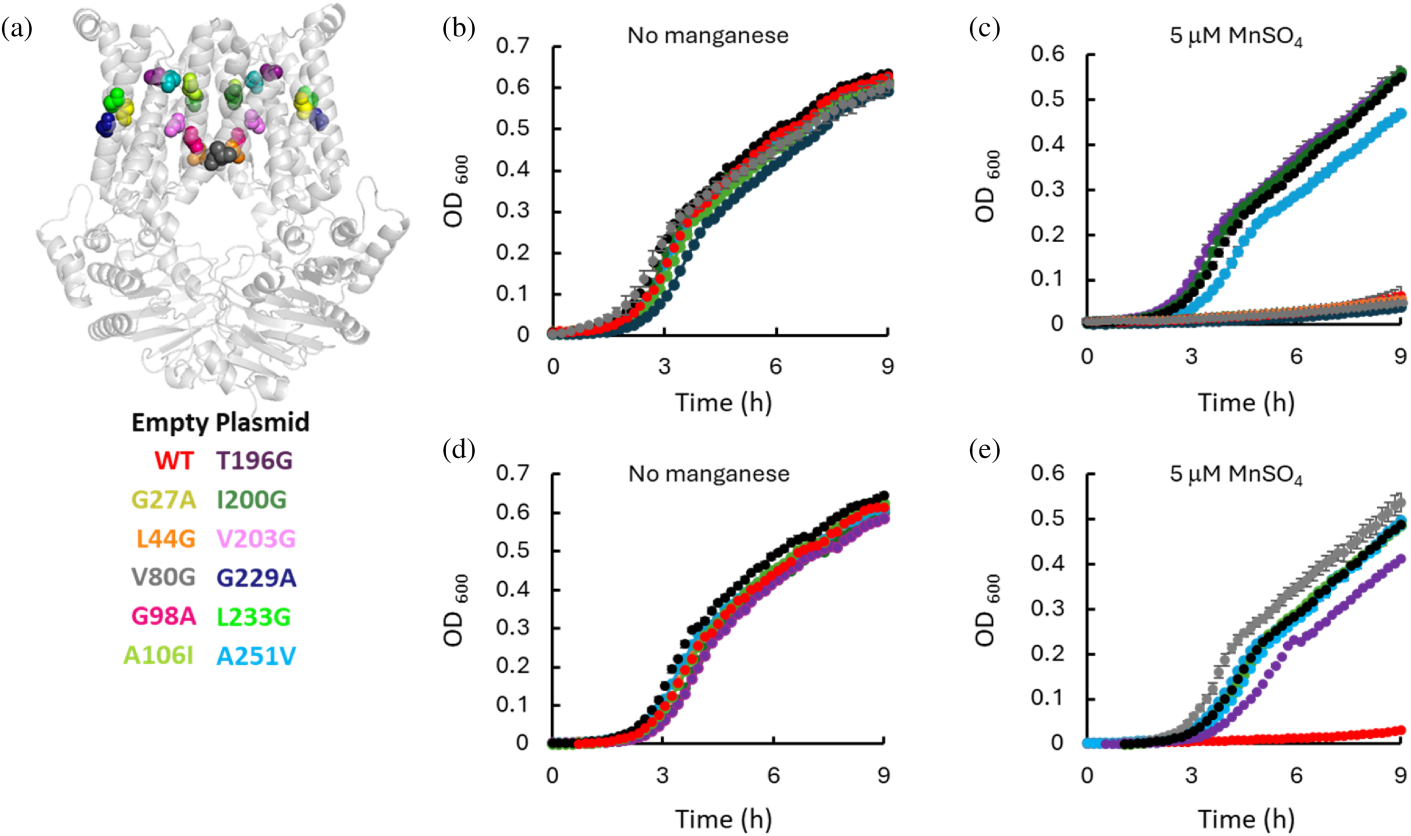
GNM‐TE successfully predicts novel functionally essential residues of MntBC. (a) Shown is the homology model of MntBC (light gray) with the residues chosen for mutational analysis shown as spheres. (b–e) Cultures of *Bacillus subtilis ΔmntR* cells transformed with the pDR111 empty plasmid or with the same plasmid encoding WT or mutant MntBC (as color indicated) were grown in LB media supplemented with IPTG in the absence (b, d) or presence (c, e) of 5 μM MnSO_4_. Shown are averages of biological triplicates, and the error bars (shown unless smaller than icons) represent the standard deviations of the mean. The data is separated for 2 pairs of panels (b & d and c & e) for clearer visualization.

To further validate the GNM‐TE calculations and assess the functional importance of the identified allosteric peaks, we introduced point mutations. Mutations were introduced by substituting small residues with larger ones and vice versa, while ensuring the avoidance of introducing charges or helix‐disrupting residues, such as proline (Figure 6). For the mutational analysis, we focused on 11 transmembrane residues located in positions that did not intuitively suggest a functional role (Figure 6a). The roles of these residues have not been previously investigated in MntBC, PsaBC, or other ABC transporters. Our computational analysis considered the complete set of the 10 slowest GNM modes, as well as a reduced subset obtained by removing the two slowest (and most dominant) modes. Five residues (L44, G98, A106, I200, A251) identified as allosteric peaks using the full set were selected for mutagenesis, and four residues (G27, T196, P209, L233), identified using the reduced subset, were chosen. Additionally, two residues with high TE × Col scores (V203 and G229) were selected due to their proximity to other allosteric hotspots (Figures 5 and S3–S5). As a negative control, we also introduced a mutation in residue V80, which has a low TE × Col score and was therefore not expected to play an allosteric role.

Prior to functional assays, the proper membrane expression of all mutants was validated, leading to the exclusion of P209A, which was not properly expressed. All other mutants exhibited normal expression levels (Figure S6).

To test if the mutations interfered with the transport activity of MntBC we utilized a cellular manganese uptake assay as previously described (Kuznetsova et al., 2021 and see Section 4). In this assay, MntBC is expressed under the control of an inducible promoter and is not subject to repression by manganese responsive transcriptional elements. In the absence of manganese, the induction of expression of a functional manganese importer has no effect on growth. However, in the presence of excess manganese, the unregulated manganese uptake activity leads to the over accumulation of intracellular manganese and subsequent growth arrest (see Kuznetsova et al., 2021). Notably, this assay only determines whether transport is normal or impaired but does not provide direct evidence of allosteric mechanisms. However, we hypothesize that mutating a residue essential for allosteric transmission will disrupt transport, whereas mutating residues that are not essential for allostery will not affect transport.

As depicted in Figure 6b,d, in the absence of manganese, the growth of control cells transformed with an empty control vector, cells expressing WT MntBC, or cells expressing the 11 mutant variant of MntBC was identical, indicating that the mutations did not affect the general fitness of the bacteria. In contrast, in the presence of manganese, 8 out of the 10 mutations introduced at positions identified as allosteric peaks abolished the activity of MntBC (Figure 6c,e). Notably, mutating a residue with a low TE × Col score (V80G) did not affect MntBC activity (Figure 6c).

**FIGURE 7:**
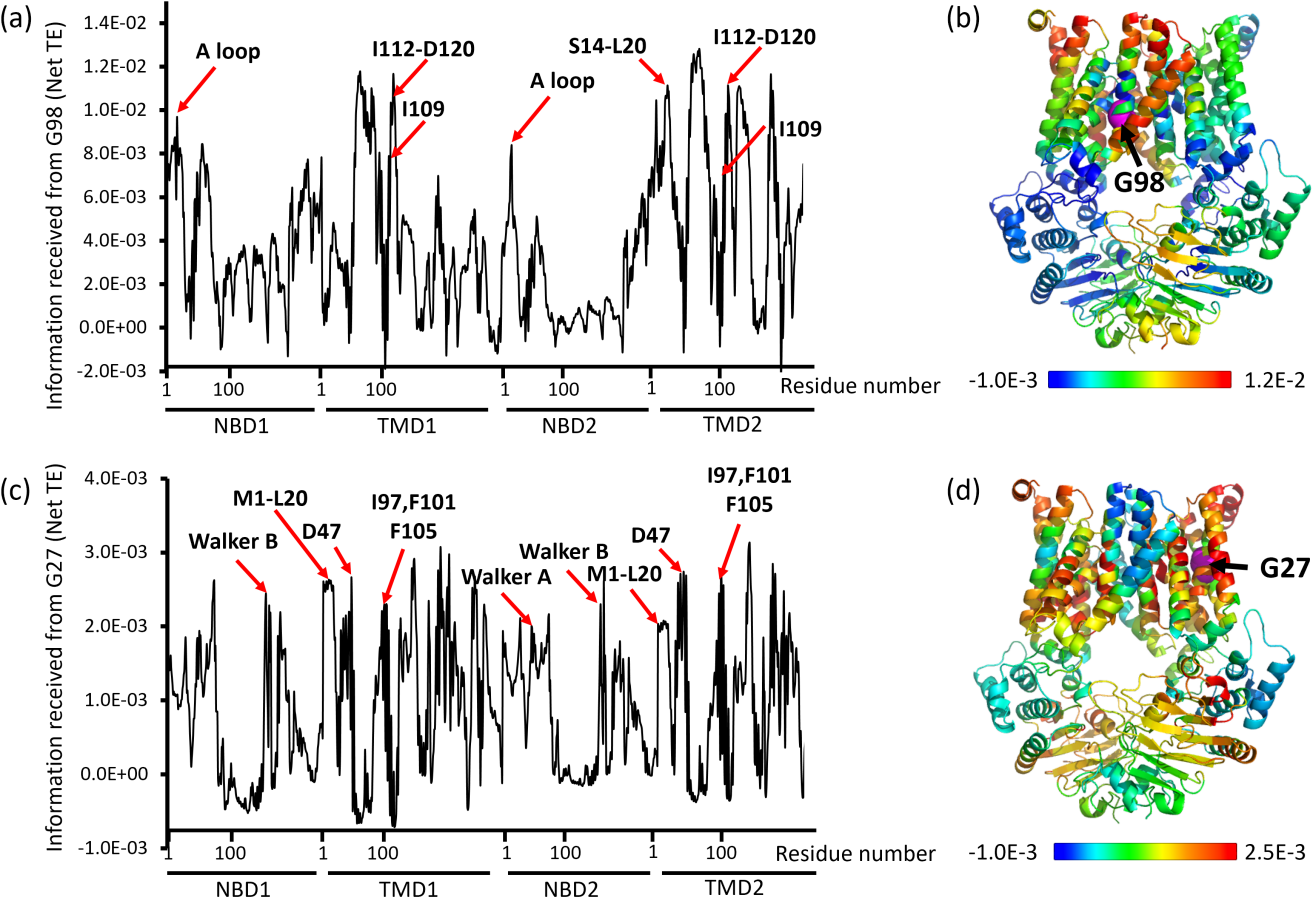
G27 and G98 allosterically control functional regions. Information received (Net TE values) by all residues of MntBC from G98 (a) or G27 (c). Red arrows indicate known functionally important sites and a site (M1‐L20) of an unknown function. Residues of MntBC are color coded according to the amount of information received (Net TE values) from G98 (b) or G27 (d), with blue and red colors denoting low and high values, respectively. G98 and G27 are colored magenta and indicated by black arrows. Net TE values were calculated using the 10 slowest modes (b) or after excluding the two slowest modes (d).

The positions of the residues identified by GNM‐TE as allosteric peaks and found to be essential for the transport activity of MntBC (Figure 6a) do not intuitively disclose their functional role. To gain insight into the mechanisms underlying the effects of such mutations we initially focused on two residues: one revealed when considering the 10 slowest GNM modes (G98) and the other (G27) identified only after excluding the two slowest GNM modes. We analyzed the domains/residues in the protein that receive information from these residues, that is, are affected by them. As shown in Figure 7a,b, G98 controls the fluctuations of residues that line the permeation pathway (including the I112‐D120 gate region), and a site of unknown function at the N‐terminus of the TMDs (residues S14‐L20). Importantly, among the residues that receive the most information from G98 are those that comprise the A loop, a region known to be essential for ATP binding (Ambudkar et al., 2006). Although G98 is relatively distant from the NBDs, it is involved in a long‐range network of allosteric interactions that affect residues within the A loop. The replacement of the flexible glycine with the more rigid alanine likely alters the conformational dynamics thereby impeding the normal allosteric signal transduction. On the other hand, the residues that receive the largest amount of information flow from G27 (Figure 7c,d) include the Walker A, Walker B, ATP‐hydrolyzing E163, a site of unknown function at the N‐terminus of the TMDs (residues M1‐L20), the manganese coordinating D47, the gating residues/residue neighbors I97, F101, and F105. Notably, all of these residues are essential for the transport activity of MntBC (Figure 6e and Kuznetsova et al., 2021). Next, we performed a similar analysis for all other positions we identified to be essential for the function of MntBC (i.e., L44, A106, T196, I200, V203, G229, L233, and A251). As shown in Figure S7A–H these allosteric hotspots predominantly influence the dynamics of the A loop and the Walker B motif at the NBDs, as well as regions within the TMDs, including the N terminus (M1‐L20), I112, and residues along the permeation pathway, many of which are known functional residues (Table S1). Notably, M1‐L20 and I112, frequently observed to be influenced by the mutation positions, are located in the extracellular regions of the TMDs. These positions could mediate the binding of the substrate (manganese) binding protein MntA.

### 2.4 The distinct dynamic footprint of metal (manganese) transporters

**FIGURE 8:**
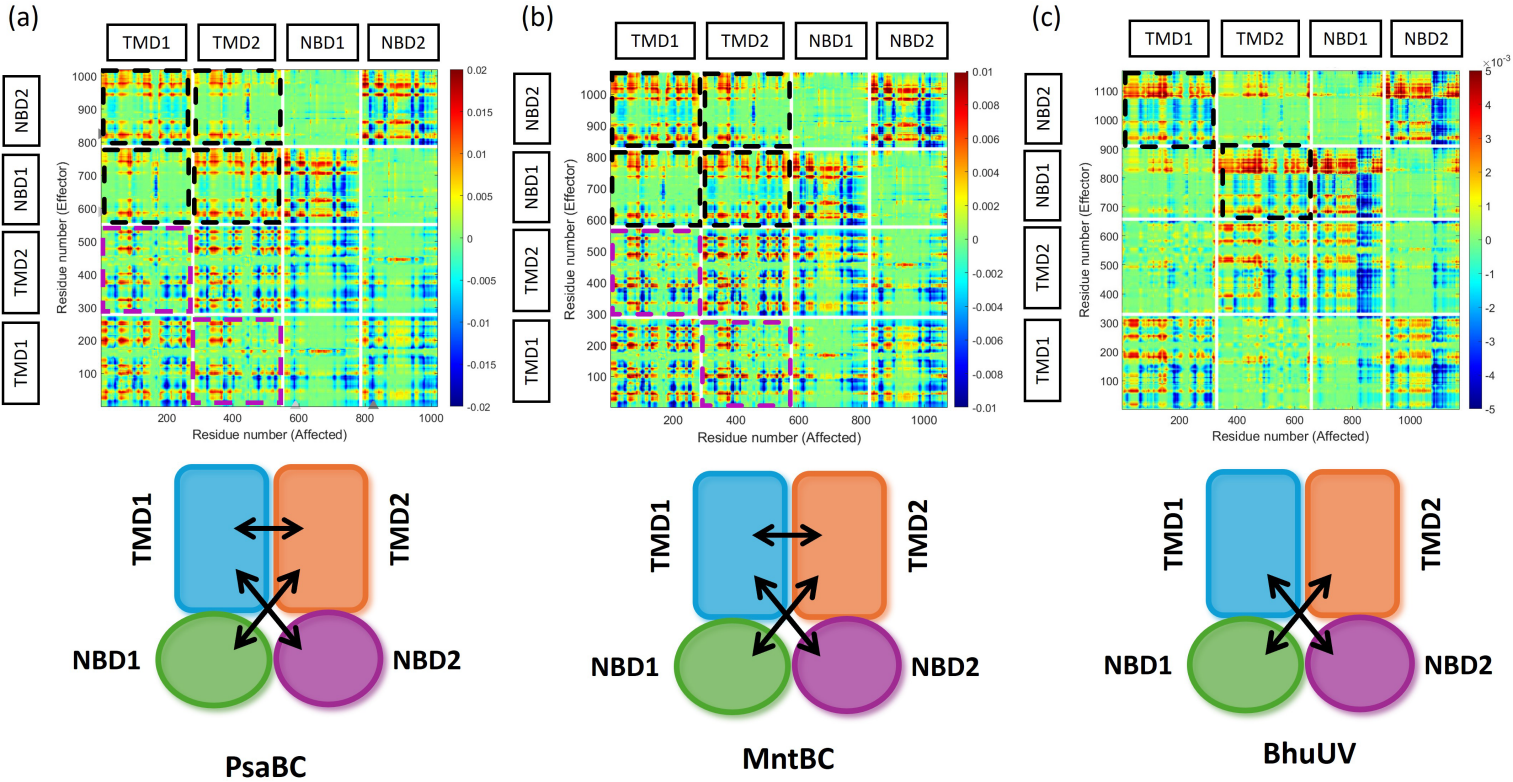
Manganese transporters employ a unique allosteric connectivity. Shown is the Net TE between all residues of the inward facing conformations of (a) PsaBC (PDB ID 7KYP), (b) MntBC (homology model), and (c) the heme importer BhuUV (PDB ID 5B57). In this 2D cross correlations map the transfer entropies (Net TE) from effector residues (Y‐axis) to affected residues (X‐axis) are color coded, with red and blue colors indicating residues that transmit high or low amounts of information, respectively. Dashed black and magenta rectangles highlight the information flow from the NBDs to the TMDs and between the TMDs, respectively. Bottom panels schematically illustrate the overall directionality of information flow in each protein.

In our analysis, we employ two parameters—collectivity (Col) and net transfer entropy (TE)—and their combined metric (TE × Col) to quantify the allosteric roles of residues and domains. Col and TE are derived from cross‐correlation maps, such as those shown in Figure 8, which also offer a broader perspective on the allosteric connectivity in PsaBC and MntBC. These maps illustrate the information sent (in terms of net TE) from effector residues (Y‐axis) to affected residues (X‐axis). As illustrated in Figure 8a,b, the 2D cross‐correlation maps of PsaBC and MntBC are very similar. This observation is expected, given the homology between the two proteins, reflected in high sequence, structure and function similarity.

Within the superfamily of ABC transporters, ABC importers of transition metals such as PsaBC and MntBC form a distinct phylogenetic branch (Srikant, 2020). Structurally, they share similarities with Type‐II ABC importers, which are responsible for importing vitamins and organo‐metal complexes (Locher et al., 2002; Naoe et al., 2016; Woo et al., 2012). The 2D cross‐correlation maps of PsaBC and MntBC bear resemblance to that of the Type‐II ABC heme importer BhuUV from *Burkholderia cenocepacia* (Figure 8c). However, although all these structures are in inward‐facing conformations, the respective maps are not identical, and a notable difference is observed: in PsaBC and MntBC, the TMs send information to one another, indicating an allosteric interaction, while in Type‐II ABC importers, such as BhuUV (Figure 8c) and BtuCD (Acar et al., 2020), they do not. This distinction may stem from the size of the cargo: Type‐II ABC importers typically transport organo‐metal complexes, characterized by their large size and numerous binding epitopes that can serve as an allosteric bridge between the TMs. Conversely, the smaller cargo of transition metal importers (a dehydrated ion) is likely less efficient in bridging the TMs, necessitating direct information transfer between them.

While the TMDs of PsaBC and MntBC engage in allosteric interaction with each other, they do not show an effective information flow to the NBDs (Figure 8a,b, bottom panels). Conversely, the NBDs exhibit the opposite behavior: they do not send information to each other but communicate with the TMDs (Figure 8a,b, top panels dashed black rectangles, and bottom panels).

In both PsaBC and MntBC, the predominant direction of information flow is from the NBDs to the TMDs, with minimal information transfer in the reverse direction (dashed rectangles in top panels of Figure 8a,b, and bottom panels of Figure 8). This pattern closely resembles observations in Type‐II ABC importers (e.g., BhuUV, BtuCD, Figure 8c and Acar et al., 2020), and it stands in contrast to the information flow observed in Type‐I ABC importers (e.g., MalFGK, see Acar et al., 2020). This dominance of allosteric signaling originating from the NBDs of PsaBC and MntBC explains experimental findings that, unlike Type‐I ABC importers, PsaBC and MntBC exhibit high rates of ATP hydrolysis even in the absence of their cargo and cognate Substrate Binding Protein (Kuznetsova et al., 2021; Neville et al., 2021).

## 3 DISCUSSION

Due to the large size and slow conformational changes of active transporters, computational studies in this field are highly challenging. As an alternative to full‐atom molecular dynamics (MD) simulations, we used an integrative approach that combines Gaussian Network Models (GNM) with transfer entropy (TE) calculations. In our analysis, we considered only the inward‐facing conformation of the transporters. It is possible that alternative conformations could exhibit different allosteric behaviors, revealing additional allosteric hotspots and links (Acar et al., 2020). However, this exploration requires new structural data beyond what is currently available.

To validate our calculations, we analyzed two homologous manganese ABC transporters and compared the computational predictions with three increasingly complex reference residue sets. The alignment between the predicted positions of functionally crucial residues and the reference residues was evaluated at three accuracy levels, considering exact matches as well as first and second coordination spheres. The results showcase the power of the GNM‐TE method, with predictions aligning extremely well with experimental data across all nine comparisons in both structures (Figures 1, 2, 5 and S2–S5). Based on GNM‐TE predictions, we generated 11 de novo point mutations and again found excellent agreement between predictions and experiments. We propose that GNM‐TE offers a computationally efficient and accessible alternative to conventional molecular dynamics approaches.

From a biological perspective, this study provides new insights into the structure–function relationships in active transporters in general, while offering specific insights into the mechanism of action of manganese ABC importers. The consensus is that the structure of a protein determines its function. While this parallelism between structure and function is fundamentally true, we report here that modest topological differences can lead to distinct allosteric connectivity networks: Both PsaBC‐A and MntBC‐A are structurally similar to type‐II ABC importers such as BtuCD‐F, FhuBD‐D, HmuUV‐T, and MolBC‐A (Locher et al., 2002; Mey et al., 2005; Pinkett et al., 2007; Qasem‐Abdullah et al., 2017). Nevertheless, despite this structural similarity, the allosteric connectivity in PsaBC‐A and MntBC‐A differs significantly from any of these transporters (Figure 8 and Acar et al., 2020). Beyond illustrating the importance of small structural changes in allostery, these results establish PsaBC‐A and MntBC‐A (and likely other ABC importers of transition metals) as a distinct group within the ABC superfamily. In light of the impending global antibiotic crisis and the clinical significance of ABC importers of transition metals (Gat et al., 2005; McDevitt et al., 2011; Remy et al., 2013), this discovery emphasizes the need for future investigations to better understand the mechanisms of this important subclass of ABC transporters.

## 4 MATERIALS AND METHODS

### 4.1 Gaussian Network Model

Gaussian Network Model utilized in this study is a minimalist 1D elastic network model (Bahar et al., 1997; Haliloglu et al., 1997). It assumes a Gaussian distribution of fluctuations that is isotropic in a protein's structural framework. This model relies on two critical parameters: the force constant, which governs harmonic interactions, and the cutoff distance that defines the range of these interactions. The force constant is uniformly set to unity for all interactions as per convention. The interaction range is determined by a cutoff distance, usually within 7 to 10 Å. In this study, a specific cutoff distance of 10 Å was utilized.

### 4.2 GNM‐TE

The integration of transfer entropy (TE) (Hacisuleyman & Erman, 2017) enhances GNM by assessing the directional flow of information between residues *i* and *j*. When residue *i* moves, TE quantifies the reduction in uncertainty regarding residue *j*'s movements by introducing a time delay τ. The formulation for *T*_*i*→*j*_ (τ) between residues *i* and *j* at time τ is:

(1) $$T_{i\to j}\left(\tau \right)=S\left(\Delta R_{j}\left(t+\tau \right)|\Delta R_{i}\left(t\right)\right)-S\left(\Delta R_{j}\left(t+\tau \right)|\Delta R_{i}\left(t\right),\Delta R_{j}\left(t\right)\right)$$

where the *S* are conditional entropies, given by:

(2) $$S\left(\Delta R_{j}\left(t+\tau \right)|\Delta R_{i}\left(t\right)\right)=\begin{array}{l} -\left\langle \ln p\left(\Delta R_{i}\left(0\right),\Delta R_{j}\left(\tau \right)\right)\right\rangle \\ +\;\left\langle \ln p\left(\Delta R_{i}\left(0\right)\right)\right\rangle \end{array}$$

(3) $$S\left(\Delta R_{j}\left(t+\tau \right)|\Delta R_{i}\left(t\right),\Delta R_{j}\left(t\right)\right)=-\left\langle \ln p\left(\Delta R_{j}\left(0\right),\Delta R_{j}\left(\tau \right)\right)\right\rangle +\;\left\langle \ln p\left(\Delta R_{i}\left(0\right),\Delta R_{j}\left(0\right),\Delta R_{j}\left(\tau \right)\right)\right\rangle$$

The net TE from residue *i* to *j* at a given τ is described as:

(4) $${\Delta T}_{i\to j}\left(\tau \right)=T_{i\to j}\left(\tau \right)-{\Delta T}_{j\to i}\left(\tau \right)$$

where Δ*T*_*i*→*j*_ (τ) determines the TE direction between residues *i* and *j* at a specific time delay τ. Positive net TE values identify entropy sources, disseminating information collectively to multiple residues. Conversely, negative net TE values in entropy receivers indicate their role in acquiring information from the structure.

The choice of τ, the time delay between residue movements, plays a crucial role in uncovering causal relationships. A small τ primarily reveals local cause‐and‐effect relations among adjacent amino acids. Conversely, a sufficiently large τ can obscure or reveal few long‐range cause‐and‐effect relationships. In our previous study (Altintel et al., 2022), we extensively explored various τ values. We found that an optimal τ, which maximizes collectivity in net TE values, often hovers around 3 times τ_opt_. Here, τ_opt_ represents the time window where the total TE of residues reaches its peak.

### 4.3 Degree of collectivity and TExCol score

The degree of collectivity in information transfer measures how much the movement of one residue affects a group of residues, showcasing the collective nature of information transfer. Inspired by Bruschweiler's research (Brüschweiler, 1995), we compute collectivity values for residues engaged in information transfer. Derived from positive net TE values, these values offer insights into how specific residues collectively influence information flow within the protein structure (Altintel et al., 2022). The collectivity value *K* of residue *i* is defined as:

(5) $$K_{i,s}=\frac{1}{N}\exp\left(-\sum_{j=1}^{N}\;\;\alpha {\left(\Delta T_{i\to j,s}\left(\tau \right)\right)}^{2}\log\left(\alpha {\left(\Delta T_{i\to j,s}\left(\tau \right)\right)}^{2}\right)\right)$$

where *s* is a selected subset of slow GNM modes, *N* is the total number of residues, and α is the normalization factor which is determined as:

(6) $$\sum_{j=1}^{N}\;a{\left(\Delta T_{i\to j,s}\left(\tau \right)\right)}^{2}=1$$

In this context, the variable “*s*” includes any subsets of the slowest modes: The time delay between movements, τ, is set at three times the optimal tau value. The TE × Col score for each residue, referred to as residue *i*, is calculated by multiplying its cumulative positive net TE value by its collectivity value *K*_*i,s*_ within each subset *s*.

(7) $${\text{TExCol Score}}_{i,s}=K_{i,s}\cdot \sum_{j=1}^{N}\;\Delta T_{i\to j,s}$$

The TE × Col score, merging TE and collectivity values, is essential in pinpointing the most functionally significant global information source residues.

In the present calculations, we initially focused on the 10 slowest modes, as they most significantly contribute to the overall protein dynamics, with allosteric interactions primarily embedded within these modes (Figure S1). However, when averaging across all 10 modes, the most dominant (i.e., the slowest) modes may mask the contributions of other, less dominant yet functionally important modes. To mitigate this averaging effect, we repeated the calculations, sequentially removing the slowest mode each time, resulting in the use of subsets 1–10, 2–10, … 5–10. We used the degree of collectivity in information transfer (K) based on the maximum entropy principle, given in Equation (7), to rank the potential of these subsets of slow modes in revealing allosteric hotspots or information sources that coordinate functional motions, that is, transfer information collectively. Our analysis showed that the 1–10, 2–10, and 3–10 subsets revealed residues with relatively higher collectivity values in information transfer, setting them apart from the other subsets (Figure 3).

To further explore the impact of different subsets, particularly for the contribution of the relatively less global (more localized modes) within the 10 slowest modes, we also considered using the five slowest GNM modes instead of the 10 slowest modes, analyzing the subsets 1–5, 2–5, and 3–5. While the overall patterns were generally preserved compared to the respective 1–10, 2–10, and 3–10 subsets, we found that the NBDs (nucleotide‐binding domains) particularly benefited from including the sixth to tenth slow modes. These additional modes enhanced the collective allosteric nature of distinct sites within the NBDs, thereby contributing to a more nuanced understanding of their functional dynamics across all three subsets.

### 4.4 Statistical analysis

The subsequent spatial correlation analysis evaluated the frequency of functionally essential residue positions, grouped into three sets by the number of functionally essential residues (Tables S1 and S2), aligning with these peaks. The assessment involved cutoff distances of 0 (exact match), 4, or 7 Å.

We assessed the statistical significance of the spatial correlation between functionally essential residues and allosteric hotspot peaks with cutoff distances of 0, 4, or 7 Å. By creating 100,000 sets of randomly placed entropy source residues, we counted how frequently functionally essential residue positions from each of the three levels appeared near these random allocations. Z scores for the predictions were calculated as:

(8) $$Z=\frac{X-\mu }{\sigma }$$

where *X* represented the count of correct predictions, and μ and σ indicated the mean and standard deviation of correct matches in random samples, respectively. Following this, *p* values for the predictions were calculated using a one‐tailed hypothesis test with a significance level set at 0.05.

### 4.5 Homology modeling

A homology model of the Mnt transporter was built using the experimentally determined structure of PsaBC from *Streptococcus pneumoniae*, identified by the PDB ID 7KYP, as a template. The process involved using chains A (ATP‐binding subunit) and B (permease subunit) of the 7KYP structure as separate templates to generate the MntB and MntC components. The sequence identity between chain A and MntB, and between chain C and MntC, were 41% and 51%, respectively. Following the modeling of MntB and MntC, two copies of each were aligned onto the full tetramer template (7KYP), constructing the tetramer structure. The coordinates and pairwise alignment files to the template are provided in Data S2.

### 4.6 Preparation of membrane fractions and expression validation

*Bacillus subtilis* Δ*mntR* cells were transformed with the pDR111 empty plasmid or with the same plasmid encoding WT or mutant MntBC (as indicated). Cultures were grown to mid log phase in LB media supplemented with IPTG. Cells were harvested by centrifugation and washed once with 50 mM Tris HCl, pH 7.5, and 0.5 M NaCl. Cell pellets were resuspended (20% w/v) in 50 mM Tris HCl, pH 7.5, 0.5 M NaCl, 30 μg/mL DNase (Worthington), one EDTA‐free protease inhibitor cocktail tablet (Roche), 1 mM CaCl2, and 1 mM MgCl2 and ruptured by three cycles of tip‐sonication. Debris and unbroken cells were removed by centrifugation (10,000 g for 20 min), and the membranes were collected by ultracentrifugation at 135,000 g for 30 min, washed once in 25 mM Tris HCl, pH 7.5, 0.1 M NaCl, and resuspended in the same buffer to 1 mg/mL. 1 μg of total membrane protein from each sample were separated by SDS‐PAGE using precast 4–20% Mini‐PROTEAN® TGX gradient gels (Biorad) and following standard Western Blotting expression of WT and mutant MntBC was visualized using an anti‐His antibody (Abcam).

### 4.7 Manganese sensitivity assay

*Bacillus subtilis* Δ*mntR* strain used in this work is from BGSC. Point mutations were introduced into WT baMntBC‐A by using the QuikChange Lightning Site‐Directed Mutagenesis Kit (Agilent Technologies), and were confirmed by sequencing. Manganese sensitivity assay was performed as previously described (Kuznetsova et al., 2021). Briefly, *B. subtilis ΔmntR* transformed with control (pDR111) or baMntBC‐A plasmids were grown in LB supplemented with 150 μg/mL spectinomycin at 37°C, diluted to OD_600_ of 0.05, and grown with 1 mM IPTG in the absence or presence of the 5 μM manganese in an automated plate reader (Infinite M200 Pro; Tecan). The absorbance of the cultures was measured every 10 min for 12 h. All assays were performed in triplicates.

## AUTHOR CONTRIBUTIONS

**Ozge Duman:** Data curation; investigation; methodology; software; validation; visualization; conceptualization; formal analysis. **Anastasiya Kuznetsova:** Conceptualization; investigation; validation; visualization; formal analysis; data curation; methodology. **Nurit Livnat Levanon:** Conceptualization; investigation; validation; visualization; formal analysis; data curation; methodology. **Moti Grupper:** Investigation; conceptualization. **Akarun Ayca Ersoy:** Investigation; methodology; formal analysis; software. **Burcin Acar:** Methodology; software. **Amit Kessel:** Conceptualization; investigation; methodology; validation; visualization; software; formal analysis; data curation. **Nir Ben‐Tal:** Writing – review and editing; project administration; supervision; funding acquisition; resources. **Oded Lewinson:** Writing – original draft; writing – review and editing; project administration; supervision; funding acquisition; resources. **Turkan Haliloglu:** Supervision; project administration; writing – review and editing; writing – original draft; funding acquisition; resources.

## CONFLICT OF INTEREST STATEMENT

The authors declare no conflict of interest.

## Supporting information


**DATA S1:** Supporting Information.

**DATA S2:** Supporting Information.

## Data Availability

The data that supports the findings of this study are available in the supplementary material of this article.
