# Supplementary material for: Computational and experimental mapping of the allosteric network of two manganese ABC transporters
Source: Protein Sci. 2025 Jan 29;34(2):e70039. doi: 10.1002/pro.70039 (PMC11779740; doi:10.1002/pro.70039)
Supplement: Supplementary file 1 — DATA S1: Supporting Information. [file PRO-34-e70039-s001.docx]

**Table S1**. Reference functional residues of PsaBC

|  | **Number of Functional Residues** | **Functional Residues** | **Description** |
| --- | --- | --- | --- |
| First Set | 4 X 2 | PsaC: D46, H50, F121, F125 | - |
| Second Set | 17 x 2 | PsaB: Y11 | A-loop |
|  |  | PsaB: A38, G39, K40 | Walker A |
|  |  | PsaB: S135, G137, Q138, R141, I144 | LSGGQ-motif |
|  |  | PsaB: D158, E159 | Walker B |
|  |  | PsaB: D165 | D-loop |
|  |  | PsaB: H191 | H-loop |
|  |  | PsaC: D46, H50, F121, F125 | - |
| Third Set | 33 x 2 | PsaB: Y11 | A-loop |
|  |  | PsaB: Q76, K77 | Q-loop |
|  |  | PsaB: G34-T42 | Walker A |
|  |  | PsaB: L134-Q138, R141, I144 | LSGGQ-motif |
|  |  | PsaB: Y154-E159 | Walker B |
|  |  | PsaB: G163, I164, D165 | D-loop |
|  |  | PsaB: H191 | H-loop |
|  |  | PsaC: D46, H50, F121, F125 | - |

**Table S2.** Reference functional residues of MntBC

|  | **Number of Functional Residues** | **Functional Residues** | **Description** |
| --- | --- | --- | --- |
| First Set | 9 x 2 | MntC: D47, H51, D94, I97, F101, F105, I109, I112 MntB:E163 | - |
| Second Set | 21 x 2 | MntB: Y14 | A-loop |
|  |  | MntB: A41, G42, K43 | Walker A |
|  |  | MntB: S139, G141, Q142, R145, L148 | LSGGQ-motif |
|  |  | MntB: D162, E163 | Walker B |
|  |  | MntB: D169 | D-loop |
|  |  | MntB: H195 | H-loop |
|  |  | MntC: D47, H51, D94, I97, F101, F105, I109, I112 MntB:E163 | - |
| Third Set | 37 x 2 | MntB: Y14 | A-loop |
|  |  | MntB: G37-T45 | Walker A |
|  |  | MntB: Q80, R81 | Q-loop |
|  |  | MntB: L138-Q142, R145, L148 | LSGGQ-motif |
|  |  | MntB: I158-E163 | Walker B |
|  |  | MntB: G167, I168, D169 | D-loop |
|  |  | MntB: H195 | H-loop |
|  |  | MntC: D47, H51, D94, I97, F101, F105, I109, I112 MntB:E163 | - |
| Mutations | 11 x 2 | MntC: G27, L44, G98, A106, T196, I200, V203, P209 (not expressed), G229, L233, A251 | - |


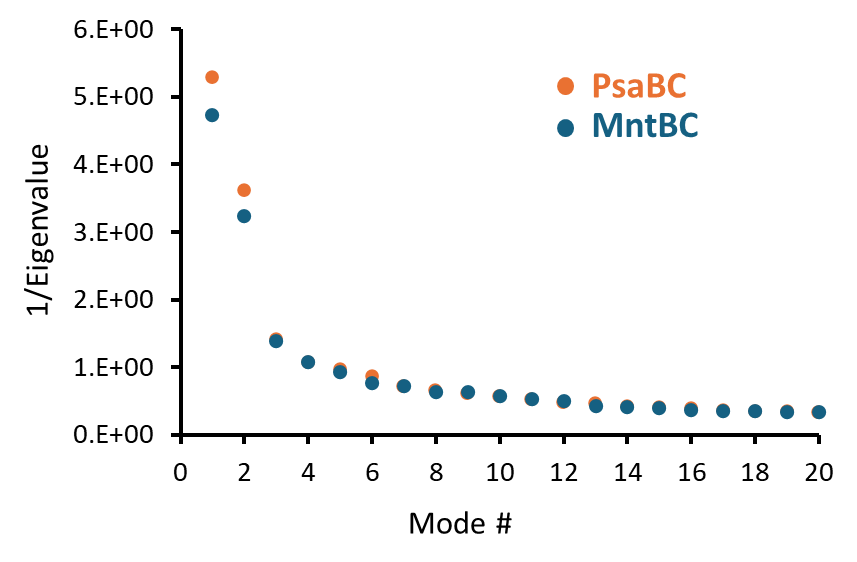


**Figure S1.** Relative contribution in 1/Eigenvalue of the slow modes of PsaBC and MntBC to the overall dynamics as a function of mode number.


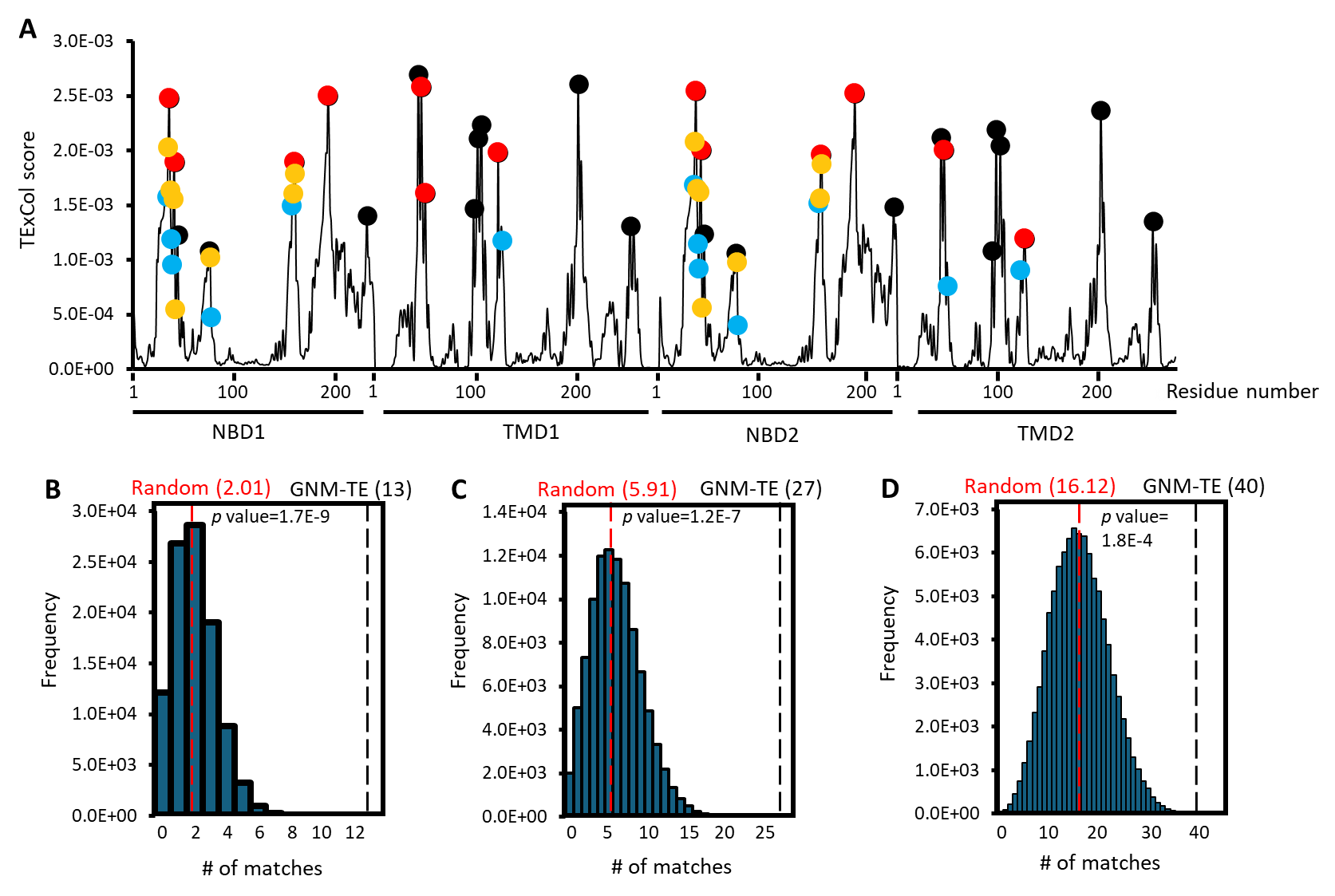


**Figure S2**. **Quality assessment of GNM-TE calculations of PsaBC with an expanded set of reference residues.** (A) Shown are the TExCol scores for all residues of PsaBC (black trace). The 31 peaks classified as allosteric peaks, are indicated by spheres. Allosteric peaks that precisely match the positions of the 66 residues comprising the third set of reference residues are indicated as red spheres and peaks that are with are within < 4Å or < 7Å are indicated as yellow and cyan spheres, respectively. (B-D) 100,000 sets of 31 randomized positions were generated and for each such set the number of matches with the positions of the expanded set of 66 reference residues was counted. Shown is the probability distribution when considering only exact matches (B), or also first-coordination sphere interactions within a cutoff distance of ≤ 4 Å (C), or also second-coordination sphere interactions within a cutoff distance of ≤ 7 Å (D). Red and black dashed vertical lines indicate the mean value of matches for the random and GNM-TE based predictions, respectively. Also shown are the p values obtained by a one-tailed hypothesis tests with a significance level of 0.05.


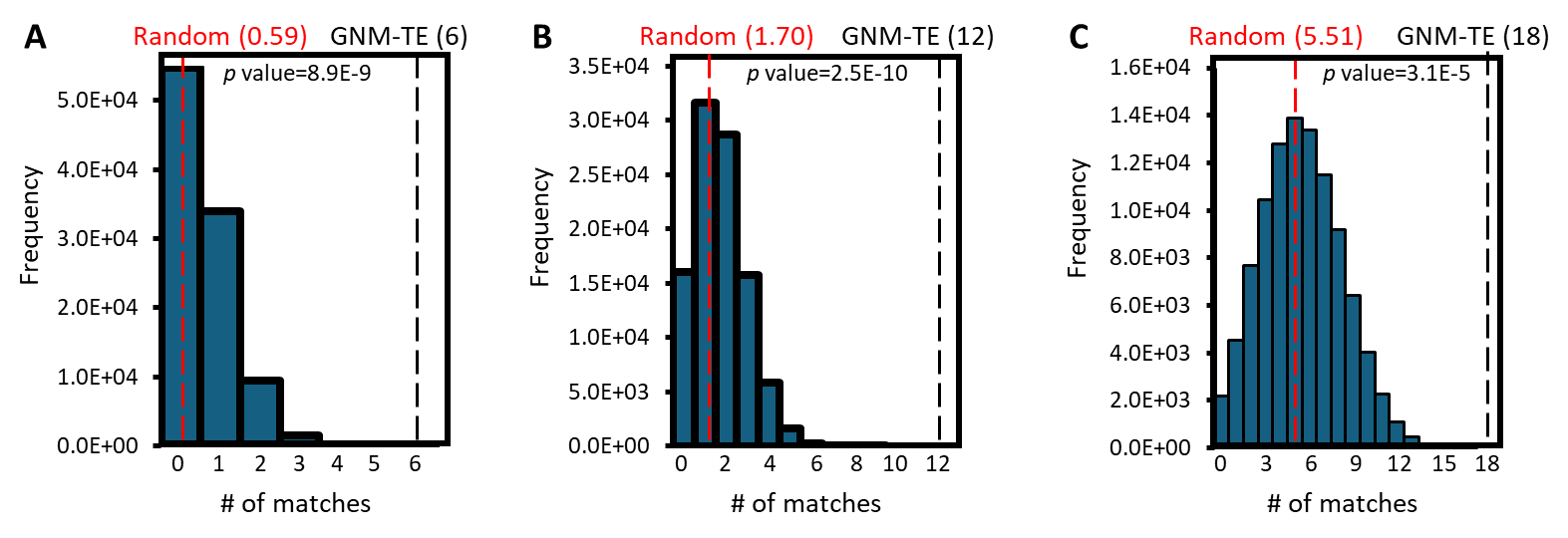


**Figure S3. Correlation between predicted allosteric hotspots in MntBC and functionally essential residues**. 100,000 sets of 35 randomized positions were generated and for each such set the number of matches with the positions of 18 essential residues was counted. Shown is the probability distribution when considering only exact matches (A), or also first-coordination sphere interactions within a cutoff distance of ≤ 4 Å (B), or also second-coordination sphere interactions within a cutoff distance of ≤ 7 Å (C). Red and black dashed vertical lines indicate the mean value of matches for the random and GNM-TE based predictions, respectively. Also shown are the p values obtained by a one-tailed hypothesis tests with a significance level of 0.05.


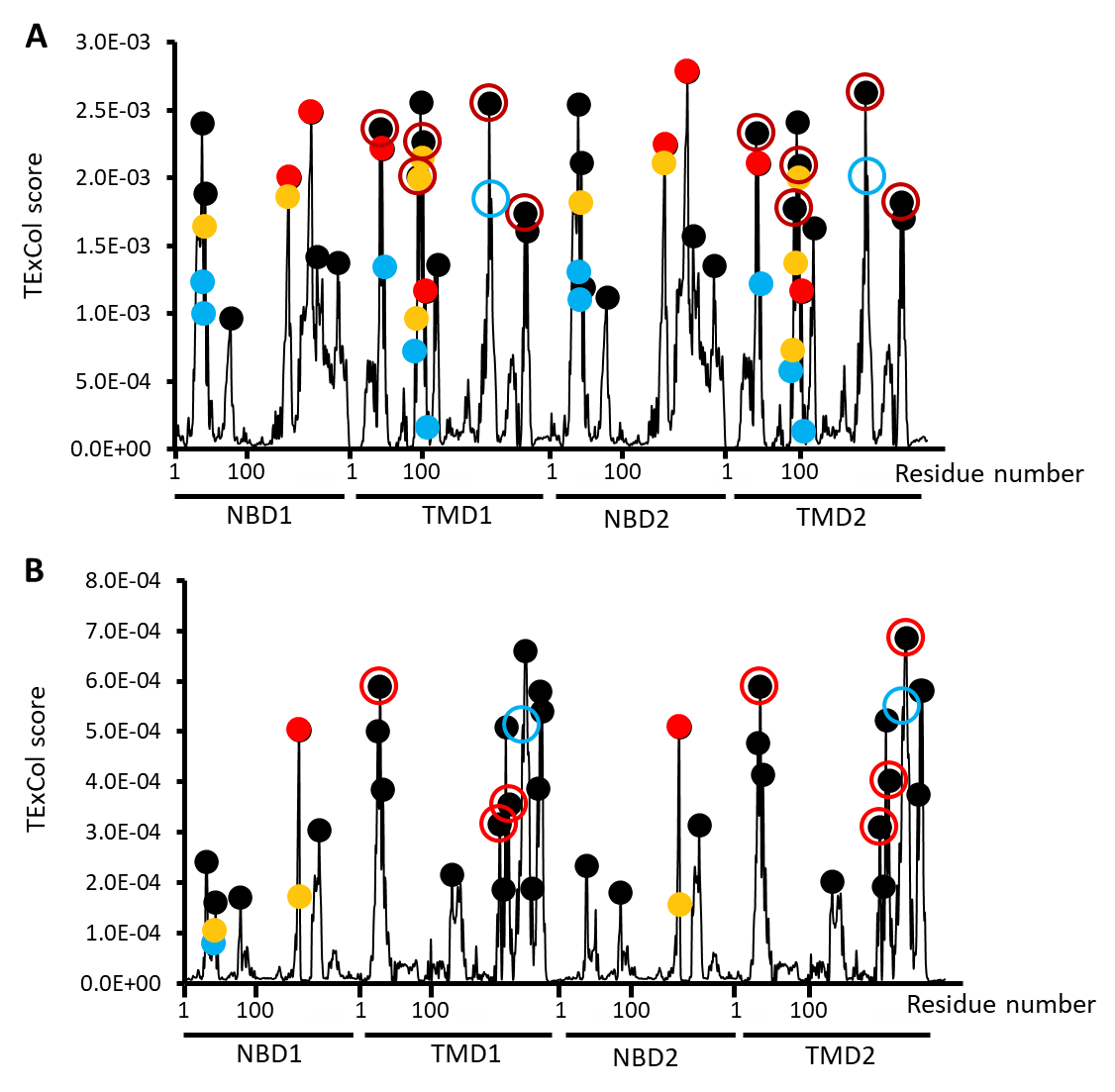


**Figure S4.** **Correlation between predicted allosteric hotspots in MntBC and the second set of functionally important residues**. Shown is the correlation between the positions of the allosteric peaks identified in MntBC and residues demonstrated to be functionally essential in MntBC or in homologous ABC transporters. Black traces indicate the TExCol scores for all residues of MntBC computed using the 10 slowest GNM modes (A) or following exclusion of the two slowest modes (B). Allosteric peaks that precisely match positions of residues comprising the second set of reference residues are indicated as red spheres, and peaks that are with are within < 4Å or < 7Å are indicated as yellow and cyan spheres, respectively. Novel functional residues predicted via GNM-TE that are exact matches with allosteric peaks or < 7Å from positions of allosteric peaks shown as open red or cyan circles, respectively.


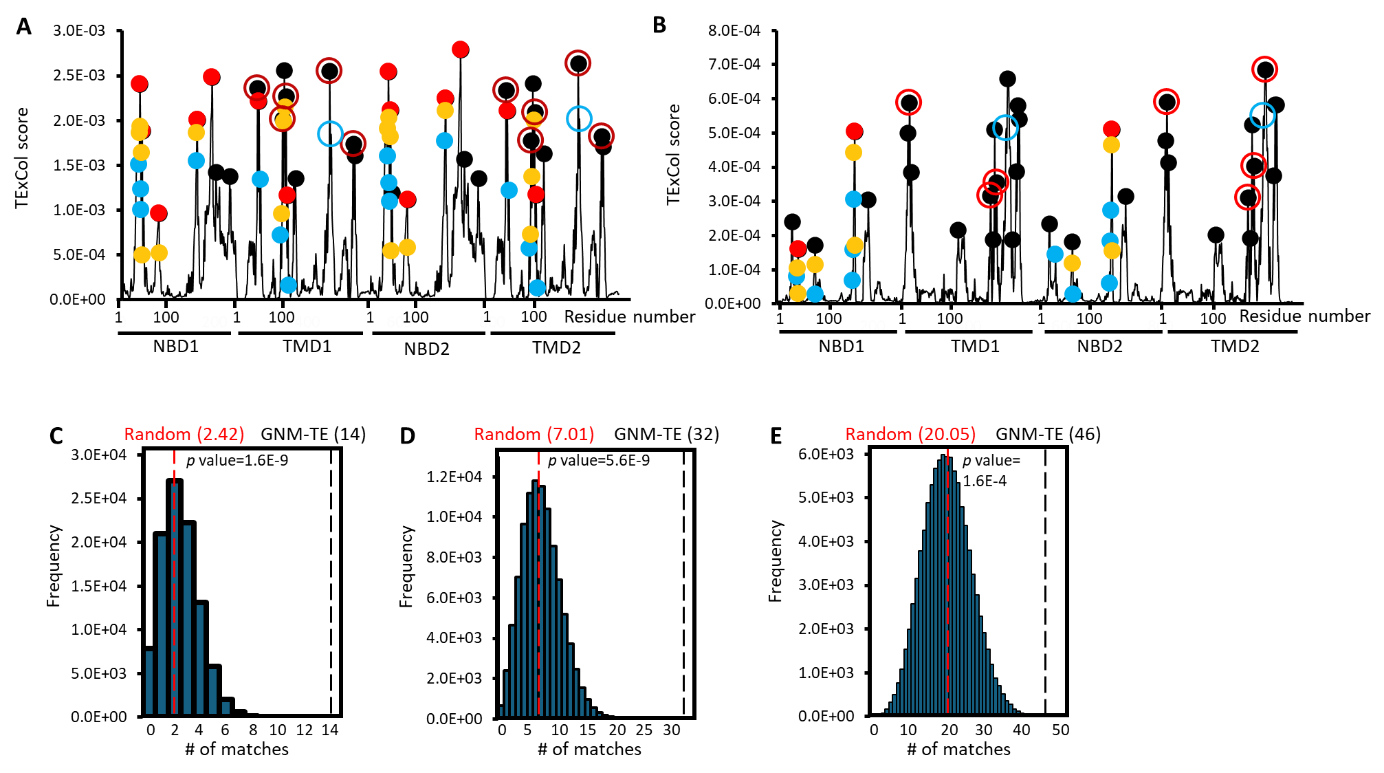


**Figure S5.** **Correlation between predicted allosteric hotspots in MntBC and the third set of functionally important residues**. Shown is the correlation between the positions of the allosteric peaks identified in MntBC and residues demonstrated to be functionally essential in MntBC, in homologous ABC transporters, and residues of the ABC transporter superfamily conserved motifs. Black traces indicate the TExCol scores for all residues of MntBC computed using the 10 slowest GNM modes (A) or following exclusion of the two slowest modes (B). Allosteric peaks that precisely match positions of residues comprising this third set of reference residues are indicated as red spheres, and peaks that are with are within < 4Å or < 7Å are indicated as yellow and cyan spheres, respectively. Novel functional residues predicted by GNM-TE that are exact matches with allosteric peaks and < 7Å from positions of allosteric peaks shown as red and cyan open circles, respectively. (C-E) 100,000 sets of 35 randomized positions were generated and for each set, the number of matches with the positions of the third set of 74 reference residues was counted. Shown is the probability distribution when considering only exact matches (C), or also first-coordination sphere interactions within a cutoff distance of ≤ 4 Å (D), or also second-coordination sphere interactions within a cutoff distance of ≤ 7 Å (E). Red and black dashed vertical lines indicate the mean value of matches for the random and GNM-TE based predictions, respectively. Also shown are the p values obtained by a one-tailed hypothesis tests with a significance level of 0.05.


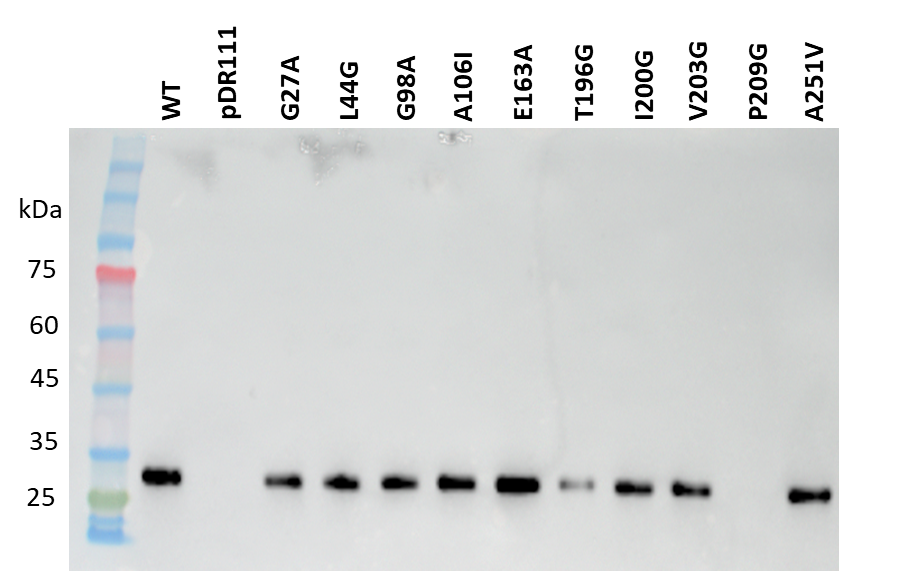


**Figure S6. Membrane fraction expression of WT and mutant MntBC**. *Bacillus subtilis* *ΔmntR* cells were transformed with the pDR111 empty plasmid or with the same plasmid encoding WT or mutant MntBC (as indicated). Cultures were grown to mid log phase in LB media supplemented with IPTG. Cells were harvested by centrifugation, and membrane fractions were prepared as detailed in the methods section. The membrane fraction expression of WT and mutant MntBC was visualized by immunoblotting of SDS-PAGE using an anti-His antibody.


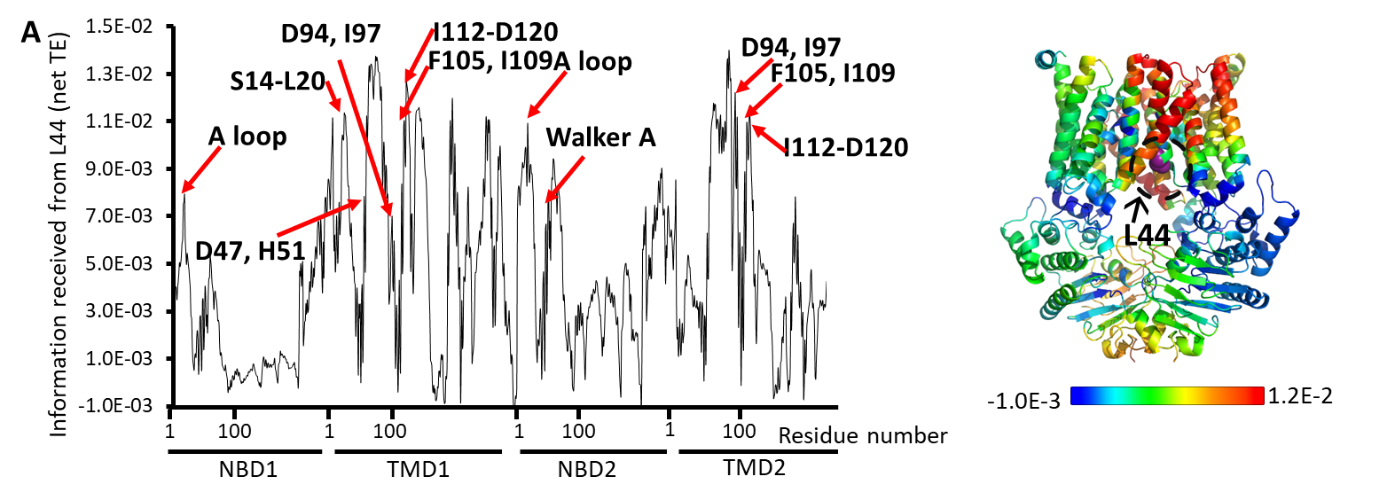


**Figure S7. Potential allosteric roles of mutated residues.**

(A) Information flow (Net TE values) from L44 to all other residues of MntBC. Red arrows indicate known functional sites and a site (M1-L20) of an unknown function (left panel). Net TE values are color-coded on the MntBC model structure, with blue and red denoting low and high values, respectively. L44 of chain B (magenta sphere) transmits information to residues of the A loop of both NBDs, Walker A of diagonal NBD, S14-L20, D47, H51 of same TMD chain, D94, I97, F105, I109 and I112-D120 of both TMDs (right panel).


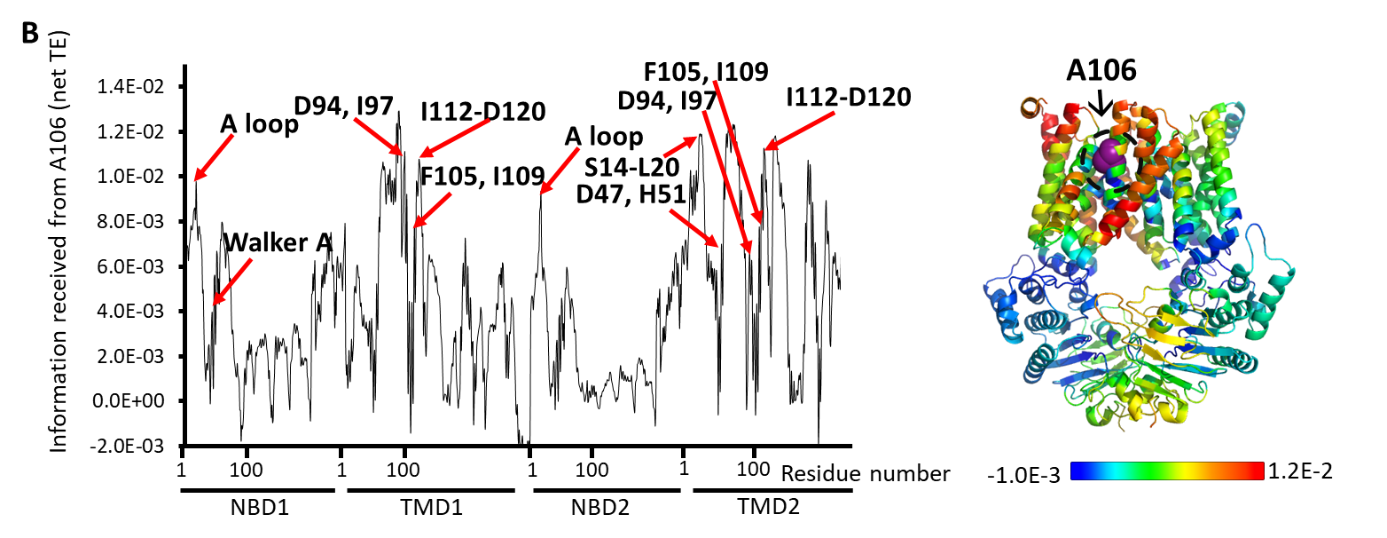


(B) Information flow (Net TE values) from A106 to all other residues of MntBC. Red arrows indicate known functional sites and a site (M1-L20) of an unknown function (left panel). Net TE values are color-coded on the MntBC model structure, with blue and red denoting low and high values, respectively. A106 of chain B (magenta sphere) transmits information to residues of the A loop of both NBDs, Walker A motif of neighboring NBD, S14-L20 of other TMD, D94, I97, F105, I109, I112-D120 of both TMDs (right panel).


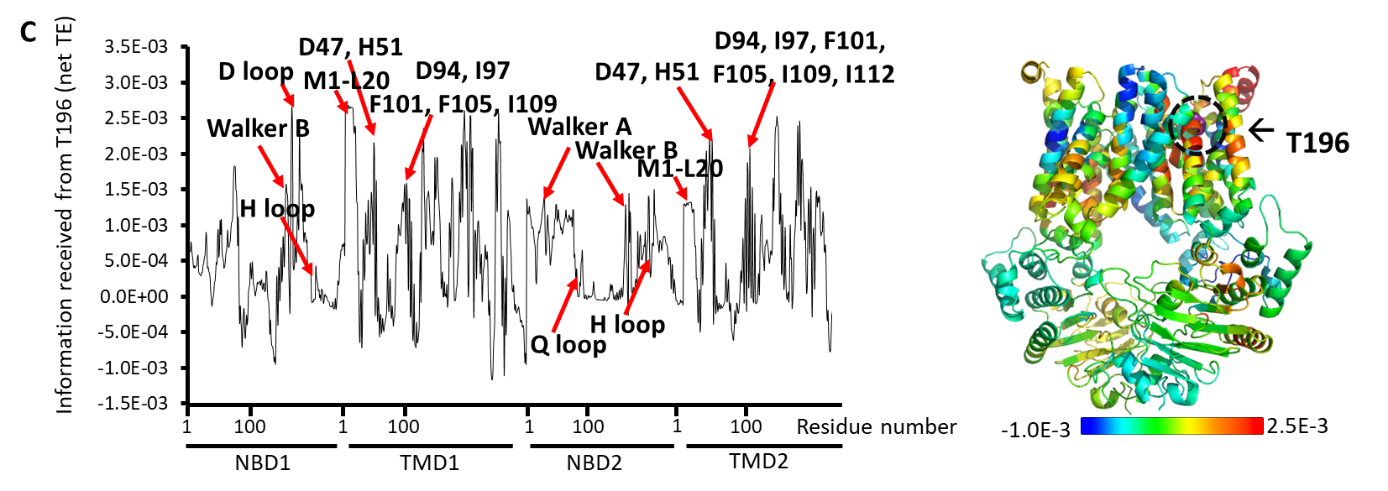


(C) Information flow (Net TE values) from T196 to all other residues of MntBC. Red arrows indicate known functional sites and a site (M1-L20) of an unknown function (left panel). Net TE values are color-coded on the MntBC model structure, with blue and red denoting low and high values, respectively. T196 of chain B (magenta sphere) transmits information to the residues/residue neighbors of the Walker B motif, H loop of both NBDs, D loop of neighboring NBD, Walker A motif and Q loop of diagonal NBD, M1-L20 D47, H51, D94, I97, F101, F105, and I109 of both TMDs and I112 of other TMD (right panel).


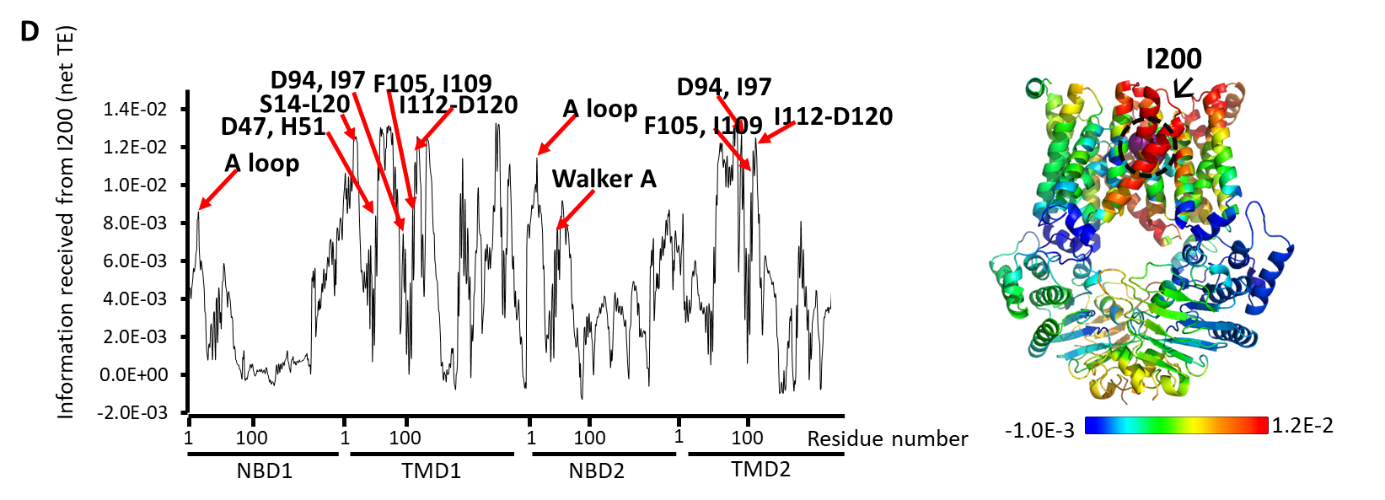


(D) Information flow (Net TE values) from I200 to all other residues of MntBC. Red arrows indicate known functional sites and a site (M1-L20) of an unknown function (left panel). Net TE values are color-coded on the MntBC model structure, with blue and red denoting low and high values, respectively. I200 of chain B (magenta sphere) transmits information to the residues/residue neighbors of the A loops of both NBDs, Walker A motif of neighboring NBD, S14-L20, D47, H51 of same TMD, D94, I97, F105, I109, and I112-D120 of both TMDs (right panel).


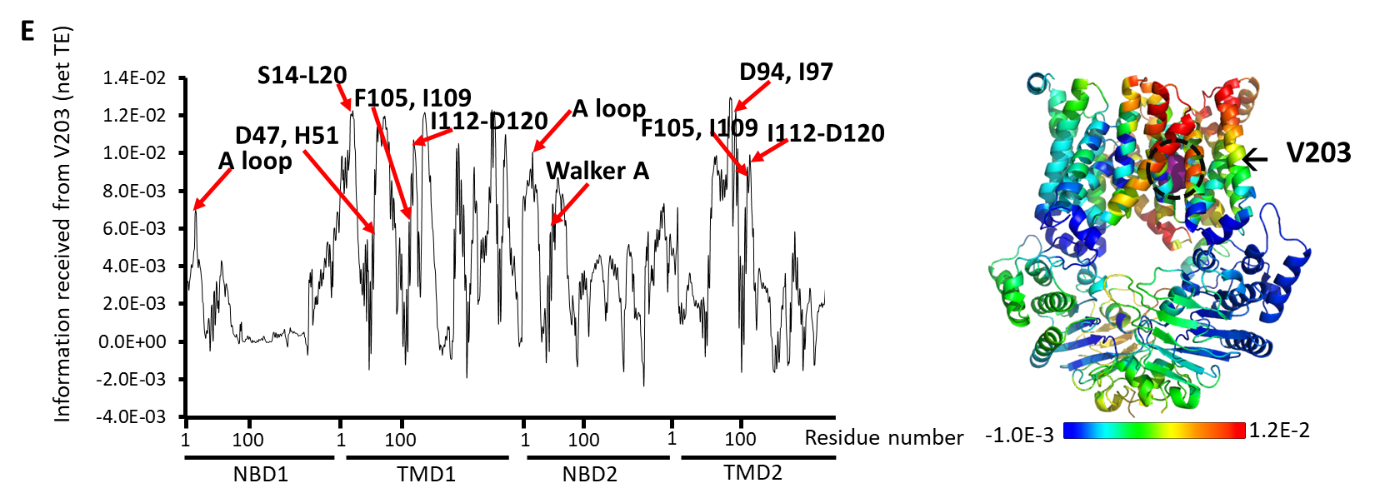


(E) Information flow (Net TE values) from V203 to all other residues of MntBC. Red arrows indicate known functional sites and a site (M1-L20) of an unknown function (left panel). Net TE values are color-coded on the MntBC model structure, with blue and red denoting low and high values, respectively. V203 of chain B (magenta sphere) transmits information to the residues/residue neighbors of A loop of both NBDs Walker A of neighboring NBD, S14-L20, D47, H51 of same TMD, D94, I97 of other TMD, F105, I109, and I112-D120 of both TMDs (right panel).


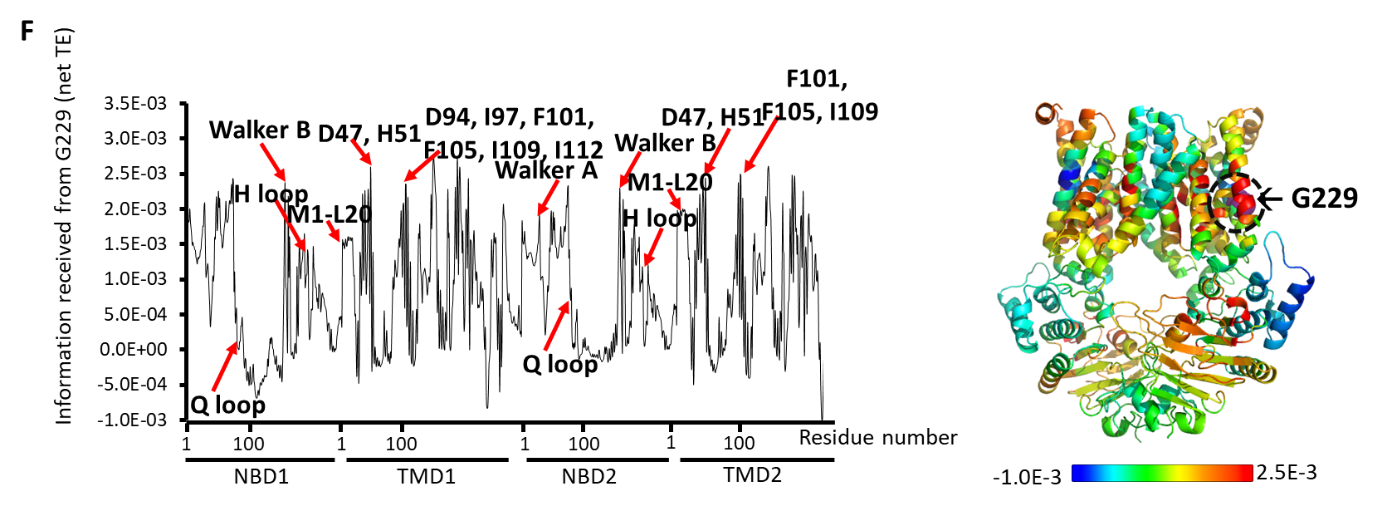


(F) Information flow (Net TE values) from G229 to all other residues of MntBC. Red arrows indicate known functional sites and a site (M1-L20) of an unknown function (left panel). Net TE values are color-coded on the MntBC model structure, with blue and red denoting low and high values, respectively. G229 of chain B (magenta sphere) transmits information to the residues/residue neighbors of Q loop, Walker B motif, H loop of both NBDs, Walker A motif of diagonal NBD, M1-L20, D47, H51, D94, I97, F101, F105, and I109 of both TMDs and I112 of same TMD (right panel).


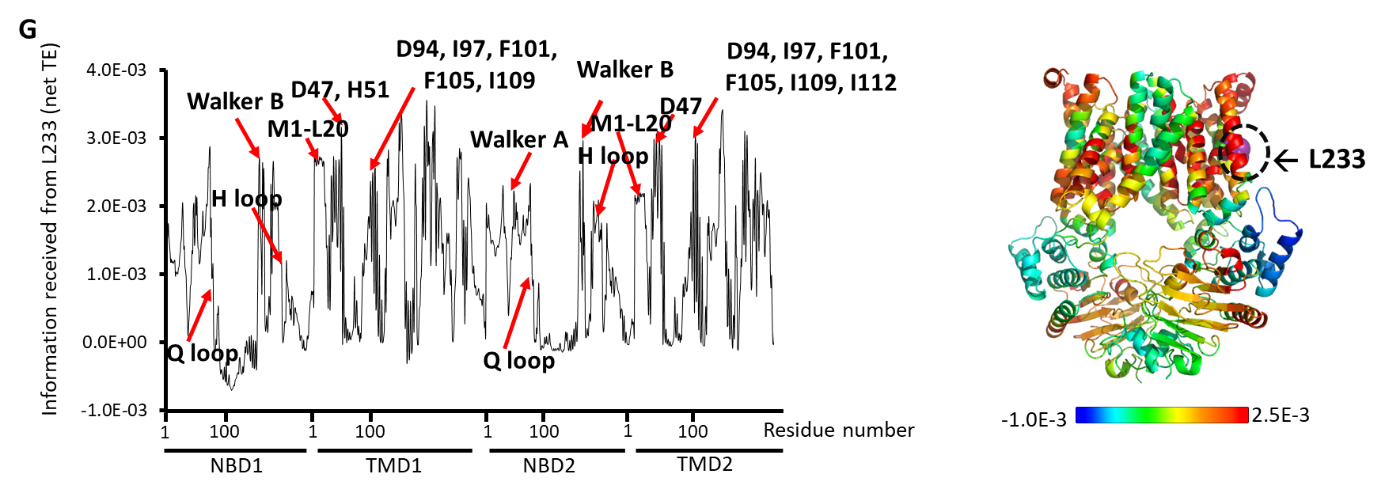


(G) Information flow (Net TE values) from L233 to all other residues of MntBC. Red arrows indicate known functional sites and a site (M1-L20) of an unknown function (left panel). Net TE values are color-coded on the MntBC model structure, with blue and red denoting low and high values, respectively. L233 of chain B (magenta sphere) transmits information to the residues/residue neighbors of Q loop, Walker B motif, H loop of both NBDs, Walker A motif of diagonal NBD, M1-L20, D47, D94, I97, F101, F105, and I109 of both TMDs, H51 of same TMD, I112 of other TMD (right panel).


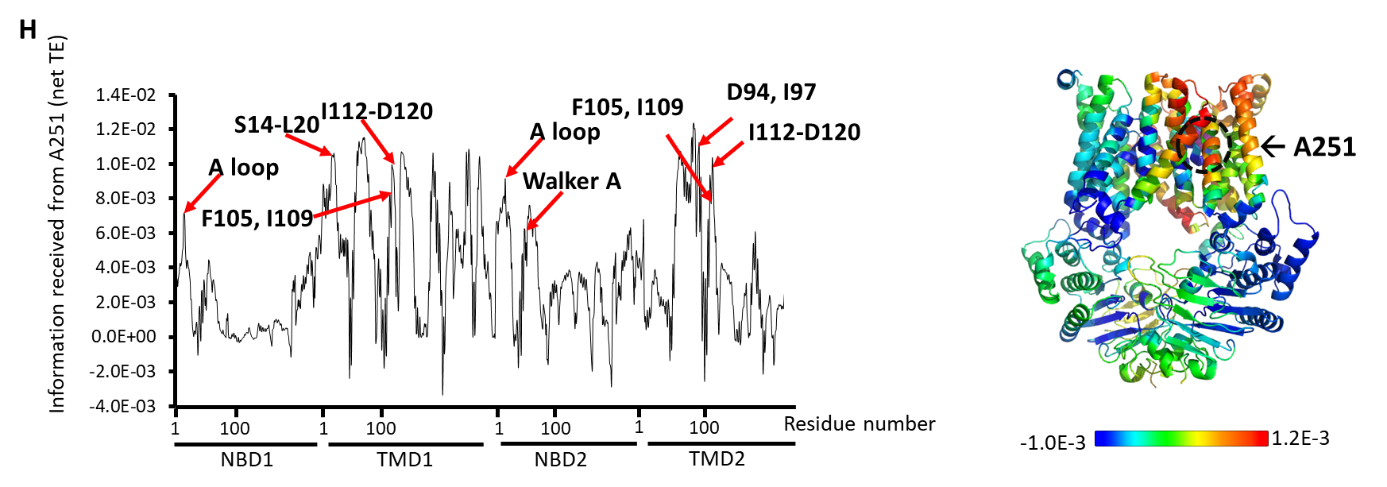


(H) Information flow (Net TE values) from A251 to all other residues of MntBC. Red arrows indicate known functional sites and a site (M1-L20) of an unknown function (left panel). Net TE values are color-coded on the MntBC model structure, with blue and red denoting low and high values, respectively. A251 of chain B (magenta sphere) transmits information to the residues/residue neighbors of A loop of both NBDs, Walker A motif of neighboring NBD, S14-L20 of same TMD, D94, I97 of other TMD, F105, I109, I112-D120 of both TMDs (right panel).
